# A novel hot exciton blue fluorophores and white organic light-emitting diodes with simplified configuration

**DOI:** 10.1038/s41598-020-62029-4

**Published:** 2020-03-20

**Authors:** Jayaraman Jayabharathi, Sekar Panimozhi, Venugopal Thanikachalam

**Affiliations:** Department of Chemistry, Annamalai University, Annamalainagar, 608 002 Tamilnadu India

**Keywords:** Materials for devices, Electronic properties and materials

## Abstract

The two efficient non-doped blue emitters with hybridized local and charge transfer state namely, NDBNPIN and DBTPIN have been synthesised and characterised. These materials are employed as a host for green and red phosphorescent OLEDs. The white device based on DBTPIN:Ir(MDQ)_2_(acac) (4%) exhibit maximum external quantum efficiency (*η*_ex_) −24.8%; current efficiency (*η*_c_) −57.1 cdA^−1^; power efficiency (*η*_p_) −64.8 lmW^−1^ with Commission Internationale de l’Eclairage (CIE:0.49, 0.40) than NDBNPIN:Ir(MDQ)_2_acac (4%) device [*η*_*ex*_ − 23.1%; *η*_*c*_ −54.6 cd A^−1^; *η*_*p*_− 60.0 lm W^−1^ with CIE (0.47, 0.42)].

## Introduction

Development of blue emitter is crucial in organic light emitting devices (OLEDs) to reduce power consumption effectively^1^. For an OLED with stable emission the current efficiency (*CE*) is proportional to external quantum efficiency (*ƞ*_*ex*_): power efficiency (*PE*) is determined by CE and operating voltage (V) [*PE* = π *CE*/V]^2–4^. Iridium and platinum based phosphorescent complexes and TADF (thermally activated delayed fluorescent materials) exhibit high *ƞ*_ex_, however, suffered with short lifetime and roll-off efficiency and also the production cost of phosphorescent materials are unfavourable for practical applications^5^. Therefore, low driving voltage with high brightness become the major issue to achieve efficient OLEDs^6^. In OLEDs, balanced hole: electron recombination leads to formation of CT exciton (charge-transfer) which undergo decay directly or relaxes to LE (local exciton), thus, utilization of both CT exciton and LE provides efficient EL (electroluminescence). From 4-(dicyanomethylene)-2-methyl-6-[4-(dimethylaminostyryl)-4*H*-pyran] with CT state maximum efficiency have been harvested^7–9^. Donor–acceptor (D–A) compounds with low % CT leads to RISC (reverse intersystem crossing) process which results high singlet utilisation efficiency(*ƞ*_*s*_), however, colour-purity is still poor due to broadened PL (photoluminescence) and EL (electroluminescence) spectra^10–13^. D-A architecture with high % LE state leads to higher efficiency because of maximum orbital overlap whereas high % CT provides low efficiency due to partial hole and electron overlap. However, because of small energy splitting (ΔE_S-T_ ≈ 0) CT state undergo RISC process results in enhanced η_s_^11,12^. These issues are overcome, by employing D–A configured emissive materials with HLCT emissive state: stabilised LE and CT states results in η_PL_(photoluminance efficiency) and high η_s._. Thus, construction of D–A emitters with HLCT emissive state is novel strategy to design efficient blue emitters.

One strategy for constructing blue emitters is the integration of high-energy emissive moieties *via* twisted arrangement which reduce the conjugation. The twisted structure with high thermal properties promotes blue emission which need for non-doped blue OLEDs^14,15^. The development of efficient host having high E_T_ (triplet-exited state energy) with good carrier transport properties is critical for efficient PhOLEDs^16^. The triplet energy of diphenylphenanthrimidazole based hosts is lower compared to individual imidazole molecule owing to enlarged π- conjugation. Therefore, tuning the molecular architecture to highly twisted molecular conformation may be an effective strategy to achieve hosts with high E_T_. In this communication, we report NDBNPIN and DBTPIN composed of phenanthrimidazole and phenyltriphenylamine with naphthyl and thienyl as spacer components used as blue emitters and host for green and red PHOLEDs.

## Result and Discussion

**Figure 1:**
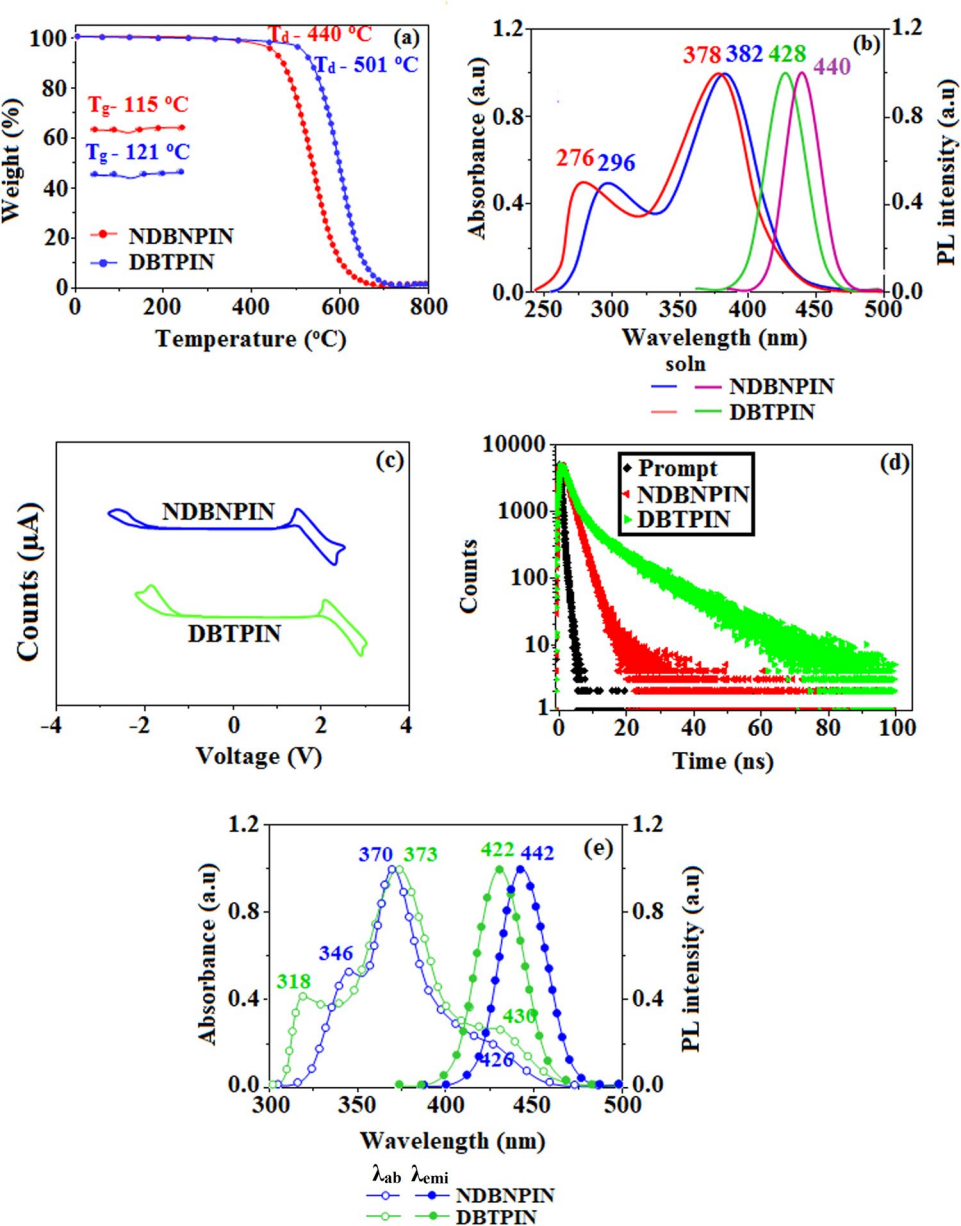
(**a**) TGA (inset: DSC) graph. (**b**) Normalized absorption and emission spectra; (**c**) cyclic voltamogram; (**d**) Life time spectra of NDBNPIN and DBTPIN and (**e**) normalized absorption and emission spectra of NDBNPIN and DBTPIN in film and solution (CHCl_3_).

The structure of the emissive materials NDBNPIN and DBTPIN are confirmed by CHN analysis, NMR and mass spectral studies. The glass transition temperature (*T*_*g*_)/*T*_*d*_ (decomposition temperature) of 115°/440° & 121°/501 °C were determined for NDBNPIN and DBTPIN, respectively (Fig. 1a). The high *T*_*d*_/*T*_*g*_ could enhance the life time and stability of OLEDs^17^. The high T_*d*_ (NDBNPIN-440 °C and DBTPIN-501 °C) indicates the high resistance of fused aromatic ring on thermolysis and high T_*d*_ could enhance the device lifetime^17^. These materials has the ability to form an amorphous glass with high glass-transition temperature (*T*_*g*_) of 115 °C – NDBNPIN and 121 °C - DBTPIN which is beneficial for the formation of stable, homogeneous and amorphous film upon thermal evaporation. Absence of endothermic peak during the measuring process reveal that no phase separation of host–guest system occur when used as host material.

**Figure 2:**
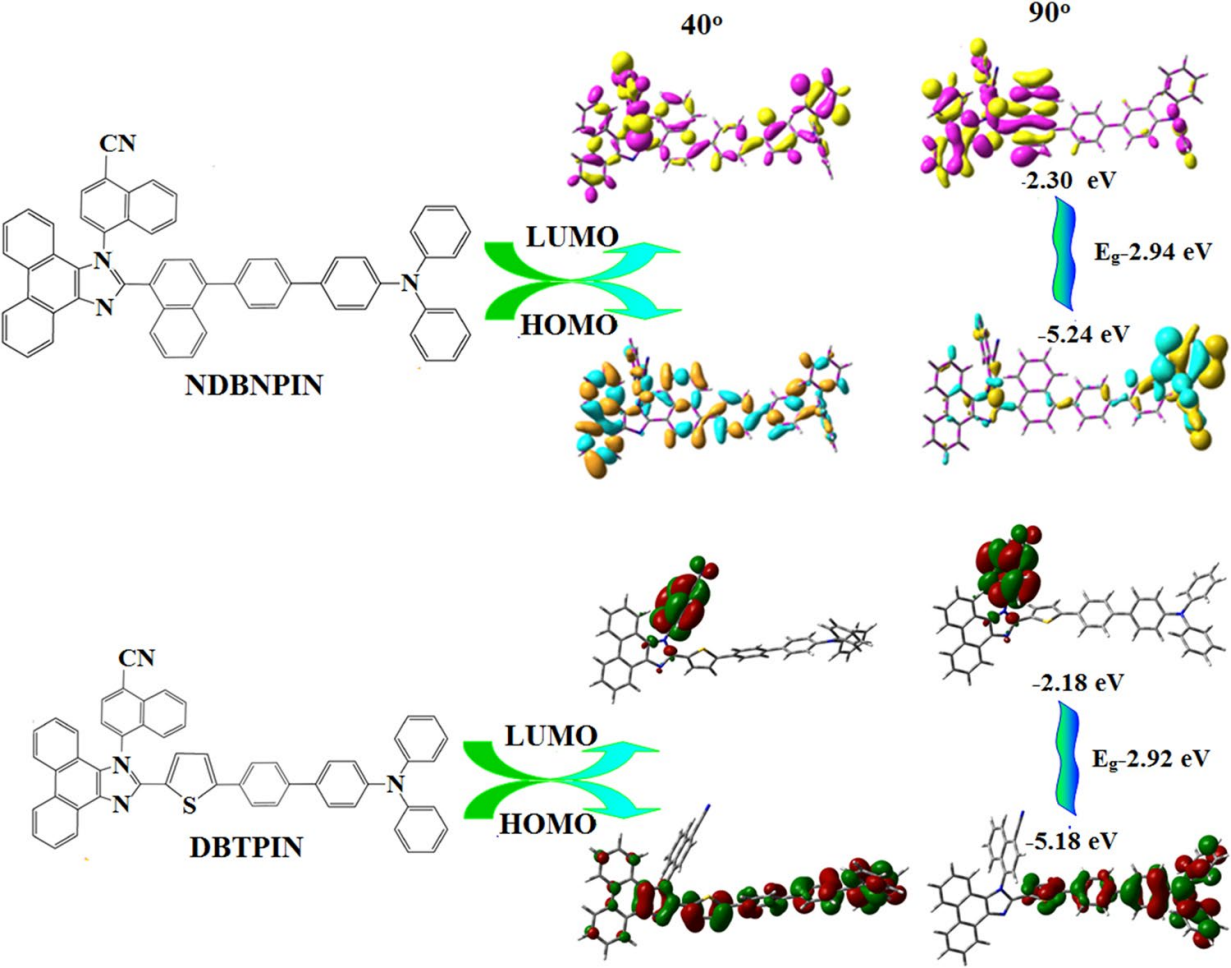
Frontier molecular orbitals and HOMO & LUMO at 40 and 90° of NDBNPIN and DBTPIN.

The onset oxidation potential of NDBNPIN and DBTPIN measured by cyclic voltammetry is 0.44 and 0.38 V (Fig. 1), respectively and their HOMO energies are 5.24 and 5.18 eV, respectively. Natural transition orbitals (HONTOs-hole- & LUNTOs-particle) of S_1_ state reveal spatial separation (CTstate), however, some orbital delocalised on entire molecule (LE) of their excited states shows presence of CT and LE components. *i.e*., HLCT emissive state. The energy of S_1_ (1.0376 eV - NDBNPIN and 1.7013 eV - DBTPIN) and T_3_ states (1.0274 eV - NDBNPIN and 1.6617 eV- DBTPIN) are almost same. A wider energy gap (E_g_) (T_3_ − T_2_/T_1_) for NDBNPIN (0.42 eV) and DBTPIN (0.57 eV) is because of same acceptor (phenanthrimidazole) group and the energy gap of DBTPIN is larger when compared to NDBNPIN. In both NDBNPIN and DBTPIN, very small ΔE_ST_ ≈ 0 facilitates RISC (T_3_ → S_1_) with hot exciton due to HLCT state. Thus, DBTPIN show high *η*_*PL*_ and high *η*_*S*_ compared with NDBNPIN. The *η*_*EQE*_ (external quantum efficiency) of device with DBTPIN is increased due to high % LE. The HOMO as well as LUMO of NDBNPIN and DBTPIN exhibit partial separation which enhanced hole- and electron-transportation (bipolar nature) with electron/hole transfer integrals, NDBNPIN (0.24/0.47 eV) and DBTPIN (0.21/0.41 eV) and minimised the ΔE_ST_ (Fig. 2).

The optical characteristics of NDBNPIN and DBTPIN were studied in solution as well as in film (Fig. 1 & Fig. S1). Absorption (λ_abs_) around 276 and 378 nm is attributed to π − π* and CT transitions, respectively and strong absorption is due to CT from triphenylamine (donor) to acceptor (naphthonitrilephenanthrimidazole)^18^. The suppressed π-π* stacking in film induced red-shifted λ_abs_ relative to solution^19^ and the larger red shift supports the CT in twisted DBTPIN or NDBNPIN. From the onset absorption in film, optical *E*_*g*_ (band gap) is calculated as NDBNPIN (2.98 eV) and DBTPIN (2.90 eV). The emitters NDBNPIN and DBTPIN show emission maxima at 440 and 428 nm, respectively (Fig. 1). As solvent polarity increased the emission spectra is red-shifted with broadened structure (Fig. S1) and exhibits larger variation in ground state dipole moment (*μ*_*g*_) relative to excited state dipole moment (*μ*_*e*_). By employing DFT and Lippert-Mataga plot (Fig. S1) *μ*_*g*_/*μ*_*e*_ was calculated as NDBNPIN (9.02/27.8 D) and DBTPIN (8.11/26.1 D). Solvents with *f* ≥ 0.2, CT state is stabilised^20–22^ (strong interaction between solvent field and CT state, LE remains unchanged) whereas solvents with *f* ≤ 0.1 LE state is stabilised. Transformation in the slope observed between butyl ether (f = 0.10) and ethyl acetate (*f* = 0.20) reveal that the emitters show HLCT emissive state *i.e*., intercrossed excited state of LE and CT [E_CT_ = E_LE_] (Fig. S1 and Table 1). The λ_emi_ of DBTPIN and NDBNPIN in film and ether is almost same due to HLCT emissive state.

**Table 1:** Optical and thermal properties of NDBNPIN and DBTPIN.

Emitters	NDBNPIN	DBTPIN
λ_ab_(nm) (soln/film)	296, 382/346, 370, 426	276, 378/318, 373, 428
λ_em_(nm)	440/442	428/430
T_*g*_/T_*d*_ (°C)	115/440	121/501
ɸ (soln/film)	80/83	90/92
HOMO/LUMO (eV)	−5.24/−2.30	−5.18/−2.26
E_g_ (eV)	2.94	2.92
τ (ns)	1.61	1.20
k_r_ × 10^8^ (s^−1^)	4.9	7.5
k_nr_ × 10^8^ (s^−1^)	1.3	0.8

The PES of NDBNPIN and DBTPIN reveal twisting of D-A linkage with 20–50° angle be the origin for intercross of CT and LE states. At 90° twist angle, frontier orbitals (HOMO and LUMO) on TPA and PPI are separated results in CT transition from HOMO (donor) → LUMO (acceptor). At 90°, twisted conformation of NDBNPIN and DBTPIN is less stable because of higher energy NDBNPIN (≈0.6 eV) and DBTPIN (≈0.04 eV) than at ≈40° (stable conformation)^20–22^. The HOMO and LUMO orbital map is displayed in Fig. 2. The high ɸ _soln/film_ (quantum yield) of NDBNPIN (83/80%) and DBTPIN (92/90%) is due to co-emission from LE and CT which is essential for efficient blue OLEDs and the enhanced quantum yield is due to decreased non-radiative (k_nr_) transition^23^ (Table 1). The oscillator strength(*f*) for λ_abs_/λ_emi_ of NDBNPIN (gas phase-372 (*f*-1.5283)/383 (*f*-1.7692); CHCl_3_-368 (*f*-1.7982)/412 (*f*-2.0462) and DBTPIN [gas phase-380 (*f*-1.5846)/392 (*f*-1.8146); CHCl_3_-370 (*f*-1.8268)/422 (*f*-1.9432)] show that oscillator strength of these compounds in CHCl_3_ is high relative to gaseous phase due to higher luminance of HLCT state in CHCl_3_ (Fig. S1). To further investigate the excited state properties, transient PL decay was recorded using time-correlated single photon counting method. The single-exponential lifetime of 1.61 ns (NDBNPIN) and 1.20 ns (DBTPIN) indicates that the hybridization of LE and CT components into a single emissive HLCT state (Fig. S1)^24–26^. The lifetime measurements in nanosecond scale further confirmed that they are fluorescent materials^22^.

**Figure 3:**
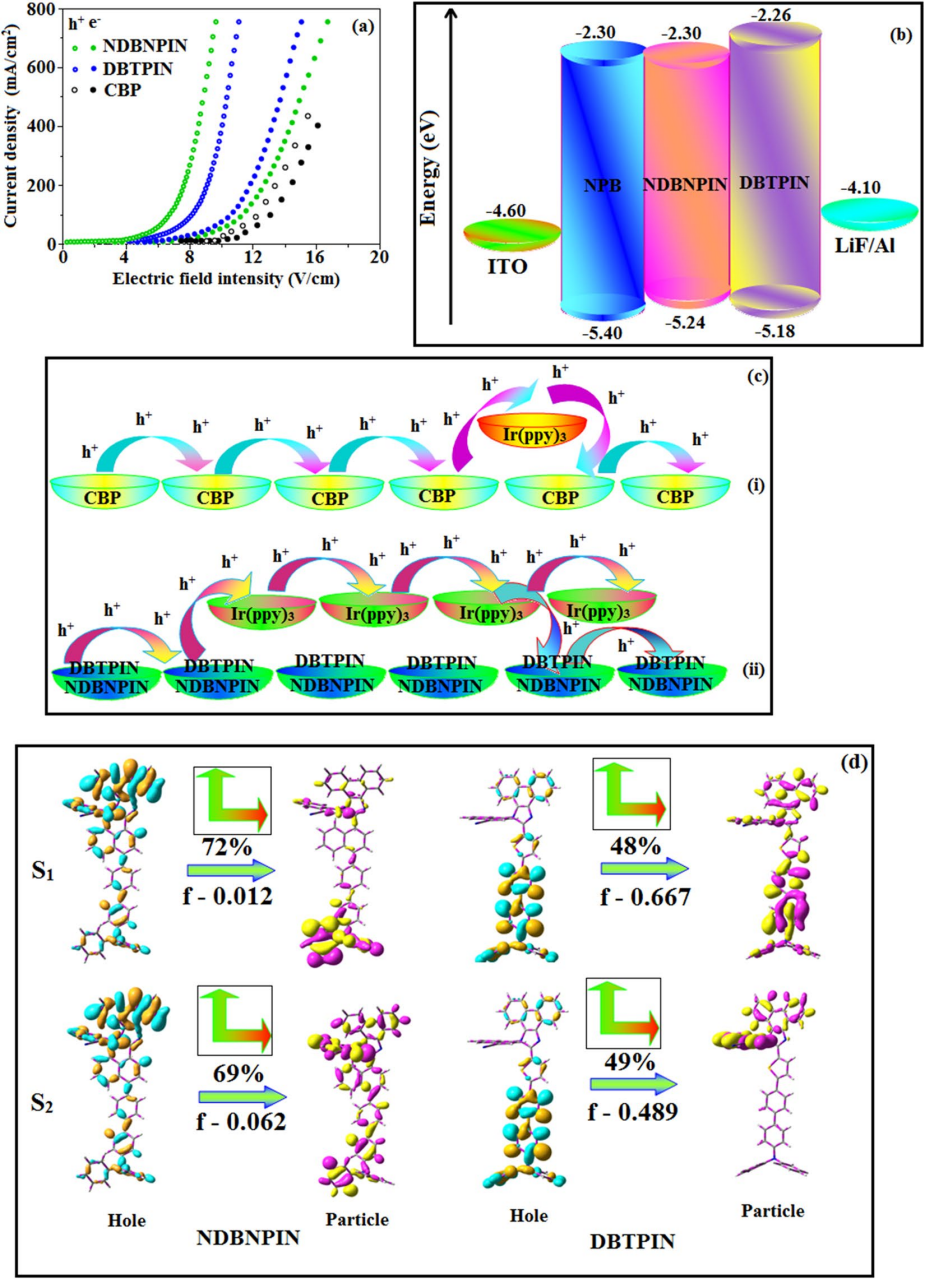
(**a**) Hole-only and electron-only devices based on NDBNPIN and DBTPIN; (**b**) Energy level diagram of non- doped devices; (**c**) Schematic representation of (i) Carrier trapping at Ir(ppy)_3,_ (ii) Carrier hopping through both Ir(ppy)_3_ and NDBNPIN and DBTPIN and (**d**) NTOs squint of NDBNPIN and DBTPIN.

The triplet energies (*E*_*T*_) estimated as 2.62 eV (DBTPIN) and 2.74 eV (NDBNPIN) which are sufficient for exciting red as well as green phosphorescent emitters. Also small ΔE_ST_ is sufficient for energy-transfer from host^27–29^ triplet to green and red emitters. The charge transportation of DBTPIN and NDBNPIN, CBP:Ir(ppy)_3_, DBTPIN:Ir(ppy)_3_ and NDBNPIN:Ir(ppy)_3_ was examined by single carrier device fabrication (Fig. 3). Current-density difference of DBTPIN and NDBNPIN compared to CBP device reveal that these bipolar-materials transport electrons and holes effectively^30^.

The carrier-current decreases in control device CBP: Ir(ppy)_3_ due to trapping at HOMO of Ir(ppy)_3_ (Fig. 3c (i)) whereas carrier current increased in DBTPIN: Ir(ppy)_3_ or NDBNPIN:Ir(ppy)_3_ devices due to direct carrier- injection into HOMO of Ir(ppy)_3_ followed by hopping transport *via* DBTPIN/NDBNPIN sites (Fig. 3c (ii)). Hole-current density of DBTPIN < NDBNPIN because cyanonaphthyl limits carrier (hole) injection/transportation significantly^12^. For devices with DBTPIN and NDBNPIN, similar electron/hole current charges were measured by high/low electric-field, respectively which shows that these materials are potential emissive candidate at low V for efficient OLEDs.

**Figure 4:**
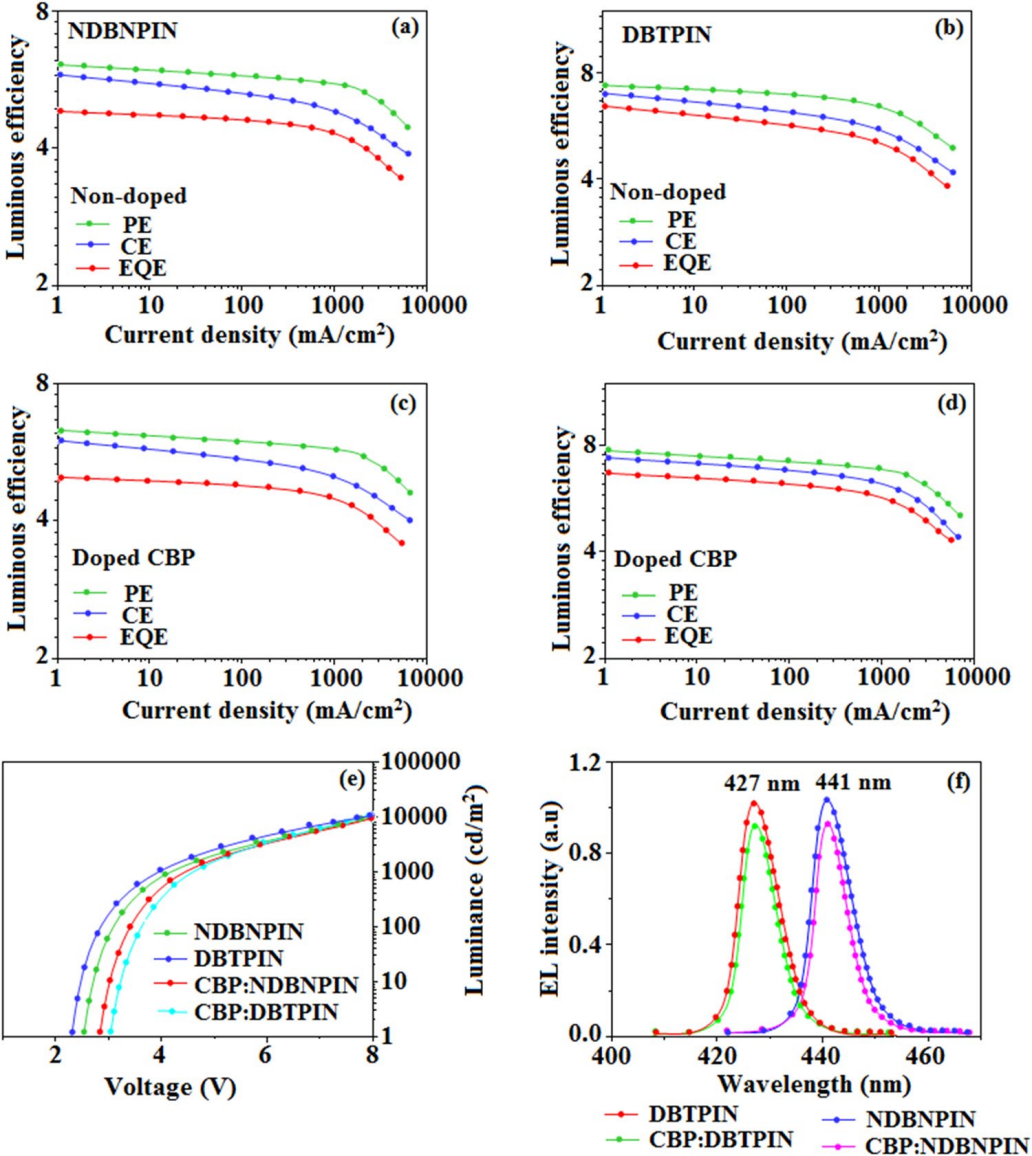
Device efficiencies: Luminous efficiency [CE (cd/m^2^), PE (lm/W), EQE (%)] -Current density (**a–d**); Luminance –Voltage (**e**); and (**f**) EL spectra of NDBNPIN and DBTPIN, CBP: NDBNPIN, CBP:DBTPIN.

The fabricated blue device, ITO/NPB (60 nm)/DBTPIN or NDBNPIN (30 nm)/LiF (1 nm)/Al (100 nm) with HOMO-LUMO energies are depicted in Fig. 3. Generally, flat-decay was shown by TADF materials because of slow TADF process in conversion of triplet state exciton → singlet state. The single-exponential decay of DBTPIN and NDBNPIN reveal that the radiative exciton are short-lived without TADF contribution and also supports single emissive state (HLCT) (Fig. 1). Therefore, high *η*_*s*_ of DBTPIN and NDBNPIN is not due to TTA or TADF process^31^. The similar PL and EL emission of DBTPIN and NDBNPIN shows that both PL and EL stems from same source with similar radiative route. The DBTPIN device exhibit superior performance (Fig. 4): (η_c_ − 7.0 cd A^−1^, η_p_ − 7.4 lm W^−1^, η_ex_ − 6.5%,CIE (0.14, 0.13)) than NDBNPIN based device (η_ex_ − 4.8%; η_c_ − 5.8 cd A^−1^; η_p_ − 6.1 lm W^−1^, CIE (0.14, 0.13)). Further, doped devices [ITO/NPB (40 nm)/TCTA(5 nm)/CBP:DBTPIN or CBP:NDBNPIN (20 nm)/TPBi (50 nm)/LiF (1 nm)/Al (100 nm): CBP-LUMO -2.7 eV: HOMO -6.1 eV] were constructed to examine the efficiencies and CBP:DBTPIN show maximum efficiencies [*η*_ex_ −6.70%; *η*_c_ −7.40 cd A^−1^; *η*_p_ − 7.8 lm W^−1^), CIE: (0.14, 0.11] (Fig. 4). Higher *η*_*ex*_ harvested from doped devices relative to non-doped one ascribed to doping concentration which reduced the exciton concentration quenching and minimised the intermolecular CT leads to bathochromic shift^32^.

The color purity of DBTPIN and NDBNPIN based devices supports that these emitters are potential candidates for full-color display. The calculated theoretical maximum *η*_*EQE*_ is of 4.6% and 4.15% [*η*_*EQE*_ = *η*_*out*_ *× η*_*rc*_ *× η*_*γ*_ *× Φ*_*PL*_^33^, ɸ_PL_: DBTPIN (92%) and NDBNPIN (83%), η_out_ - out-coupling efficiency (20%), η_rc_ - product of charge recombination efficiency (100%), *η*_*γ*_ - radiative exciton-production (25%)] and the experimental *η*_*EQE*_ is of 6.50% and 4.8%, respectively. Experimental *η*_*EQE*_ > Theoretical *η*_*EQE*_ because larger triplet exciton are converted to singlet exciton in EL process^34–36^. The *η*_r_ calculated for DBTPIN (31–38%) and NDBNPIN (25–32%) indicates γ ˂ 100% because of poor unbalanced carrier transportation in the emissive layer. Enhanced *η*_*IQE*_ 25.2% (NDBNPIN); 32.5% (DBTPIN) and maximum *η*_*s*_ 31.6% (NDBNPIN) and 38.2% (DBTPIN) [*η*_*s*_ = *η*_*out*_ × *η*_*PL*_ × *η*_*res*_ ÷ *η*_*EL*_] is because of retained CT % due to CN group in D-_Π_-A compounds. Maximum *η*_s_ breaking 25% limit: 6.6% (NDBNPIN) and 13.2% (DBTPIN) of triplet exciton converted to singlet exciton by RISC and remaining follow non-radiative process leads to high efficiency blue OLEDs. The *η*_c_ and *η*_p_ of DBTPIN device (7.0 cd/A; 7.4 lm/W) and NDBNPIN device (5.8 cd/A; 6.10 lm/W) are larger relative to TPA-PA (1.16 cd/A; 0.65 lm/W), TPA-NzP (1.00 cd/A; 0.77 lm/W) and *m*TPA-PPI (0.84 cd/A; 0.48 lm/W) devices. The quantum yield of DBTPIN (92%) and NDBNPIN (83%) is larger when compared with (i) Cz-BzP (69.7%) and TPA-BzP (49.2%) (ii) CBI (21%) and MCB (24%) and (iii) PPI-*p*CNCz (54%). Thickness of LBPPI influences *η*_c_ (50 nm: 0.01 cd/A; 40 nm − 0.13 cd/A; 30 nm: 0.40 cd/A and 20 nm: 0.68 cd/A). The *η*_c_ harvested in the current study with 30 nm DBTPIN (7.0 cd/A) and 30 nm NDBNPIN (5.8 cd/A) is higher than reported *η*_c_ (Tables S1–S3). Thickness tuning of emissive layer enhanced DBTPIN and NDBNPIN efficiencies which also supports that these materials are the best fluorescent materials. These experimental results reveal that currently non-doped [DBTPIN and NDBNPIN] and doped [CBP:DBTPIN and CBP:NDBNPIN] devices are the efficient one. Additional triplet exciton is utilized in the OLEDs because of HLCT of DBTPIN and NDBNPIN as showing the accuracy for our molecular- design- strategy.

**Figure 5:**
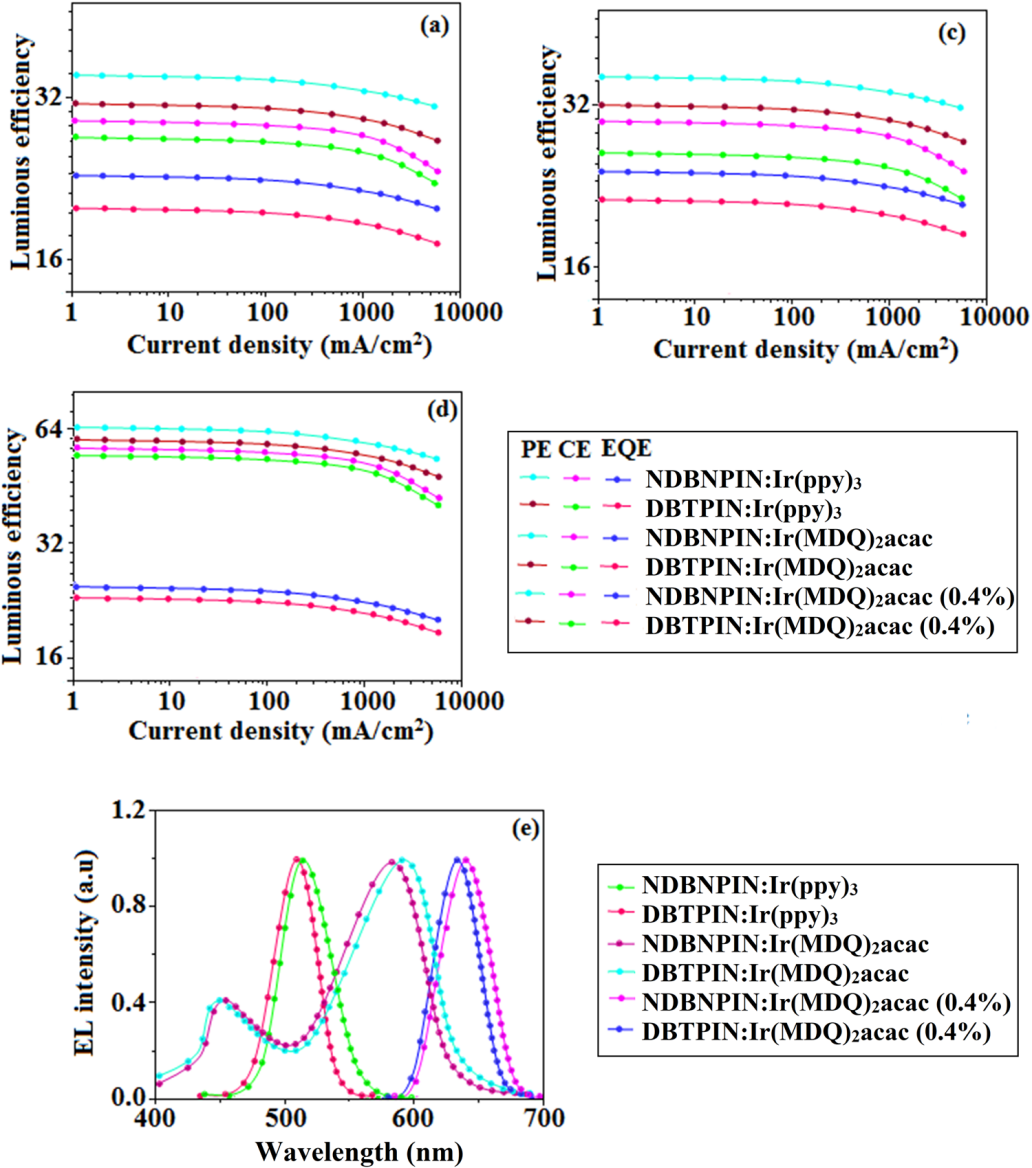
Device efficiencies: Luminous efficiency [CE (cd/m^2^), PE (lm/W), EQE (%)] - Current density (**a–c**) and Luminance –Voltage (**d**); EL spectra of green devices based on NDBNPIN:Ir(ppy)_3_, DBTPIN:Ir(ppy)_3_ and red devices based on NDBNPIN: Ir(MDQ)_2_(acac), DBTPIN:Ir(MDQ)_2_(acac) and white devices based on NDBNPIN: Ir(MDQ)_2_(acac) (0.4%), DBTPIN:Ir(MDQ)_2_(acac) (0.4%).

We have fabricated green and red PHOLEDs with configuration: ITO/NPB (40 nm)/TCTA (5 nm)/DBTPIN (30 nm): 5 wt % Ir(ppy)_3_ or NDBNPIN (30 nm): 5 wt % Ir(ppy)_3_/TPBi (50 nm)/LiF (1 nm)/Al (100 nm)]: ITO/NPB (40 nm)/TCTA (5 nm)/DBTPIN (30 nm): 8 wt% Ir(MDQ)_2_(acac)/NDBNPIN (30 nm): 8 wt% Ir(MDQ)_2_(acac)/TPBi (50 nm)/LiF (1 nm)/Al (100 nm), Ir(ppy)_3_-*fac*-tris(2-phenylpyridine) iridium(III) and Ir(MDQ)_2_(acac)-bis(2-methyldibenzo-[*f,h*] quinoxaline) acetylacetonate iridium(III) are emissive layers for green and red devices, respectively]. The device performances are presented in Fig. 5. Two emission peaks are observed at 4% Ir(ppy)_3_: part of the exciton transferred to Ir(ppy)_3_ triplet state and part of exciton transferred to ground state (S_o_) to generate phosphorescence and fluorescence, respectively^32^. At 5% doping concentration all the exciton were transferred to Ir(ppy)_3_ to generate phosphorescent emission (Fig. 5).

**Figure 6:**
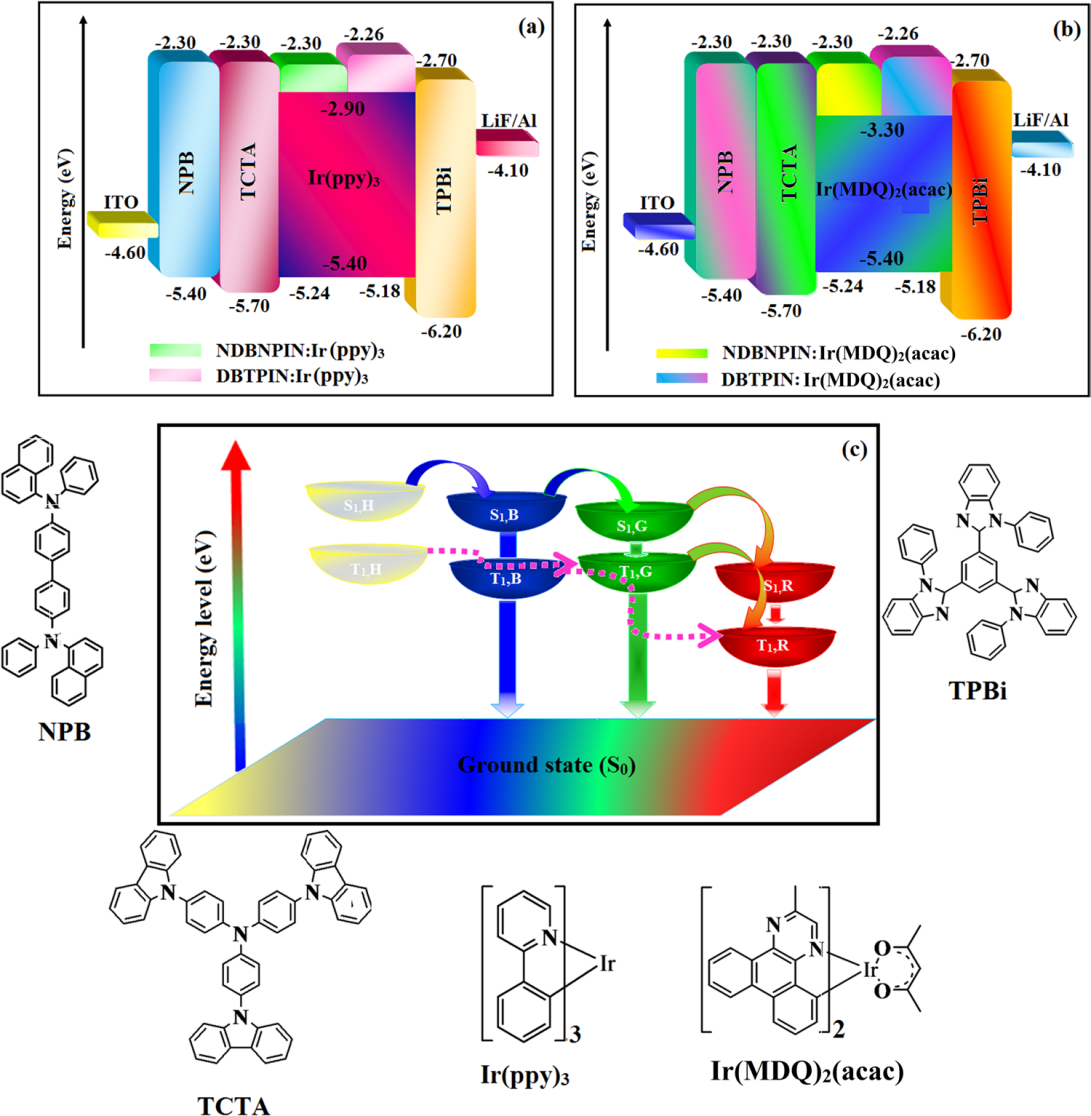
Energy level diagram of (**a**) green, (**b**) red white devices with molecular structures of functional materials and (**c**) Molecule energy levels and energy-transfer diagrams.

The green device DBTPIN (30 nm): 5 wt% Ir(ppy)_3_ shows maximum efficiency: η_c_ of 29.0 cd A^−1^ and η_p_ of 35.4 lm W^−1^ at 2.6 V; η_ex_ of DBTPIN: Ir(ppy)_3_ and NDBNPIN:Ir(ppy)_3_ are of 23.0 and 20.1%, respectively. Red device with DBTPIN: Ir(MDQ)_2_(acac) exhibits excellent efficiencies (*η*_ex_ − 24.1%; *η*_c_ − 29.8 cd A^−1^; *η*_p_ − 36.0 lm W^−1^) with CIE (0.64, 0.36) on comparison with NDBNPIN based device (*η*_ex_ − 21.3%; *η*_c_ − 26.0 cd A^−1^; *η*_p_ − 31.9 lm W^−1^) with CIE (0.64, 0.36). The enhanced efficiencies reveal that DBTPIN and NDBNPIN are the best host-materials for green and red PHOLEDs. At 0.4% doping concentration both devices based on DBTPIN: Ir(MDQ)_2_(acac) [448 nm (sh)&590 nm] or NDBNPIN: Ir(MDQ)_2_(acac) [450 nm (sh)&582 nm] show two peaks leads to white emission (Fig. 5): part of the exciton transferred to Ir(MDQ)_2_(acac) triplet state to excite phosphorescence and another part of the exciton transferred to ground state to produce fluorescence. Increasing the doping concentration, generated exciton were completely transferred to Ir(MDQ)_2_(acac) which results in phosphorescent emission. The white device based on DBTPIN:Ir(MDQ)_2_(acac) exhibit maximum *η*_ex_ − 24.8%; *η*_c_ − 57.1 cd A^−1^; *η*_p_ − 64.8 lm W^−1^ with CIE (0.49, 0.40) than NDBNPIN: Ir(MDQ)_2_(acac) based device [*η*_ex_ − 23.1%; *η*_c_ -54.6 cd A^−1^; *η*_p_ − 60.0 lm W^−1^ with CIE (0.47, 0.42)]. At doping 0.4% concentration, blue emission of the EL spectrum of NDBNPIN: Ir(MDQ)_2_(acac) device was broader than DBTPIN:Ir(MDQ)_2_(acac). More blue fluorescence contribution in device NDBNPIN: Ir(MDQ)_2_(acac) than device DBTPIN: Ir(MDQ)_2_(acac) leads to lower efficiencies of NDBNPIN:Ir(MDQ)_2_(acac) than NDBNPIN: Ir(MDQ)_2_(acac). Outstanding efficiencies indicates, DBTPIN and NDBNPIN are potential host materials and transfer of exciton is shown in Fig. 5. A part of host singlet exciton transferred to singlet blue fluorophore (BS_1_) exhibits deep-blue emission whereas another part of singlet exciton transferred to singlet green/red phosphorescent emitters (GS_1_/RS_1_) and then delivered to triplet green and red phosphorescent emitters (GT_1_/RT_1_) by intersystem-crossing process and shows red and green phosphorescent emission. Furthermore, host triplet exciton (HT_1_) is transferred to GT_1_/RT_1_ to enhance the exciton utilization (Fig. 6).

## Conclusion

We have reported two deep blue emitting materials DBTPIN and NDBNPIN with dual charge transport properties and exhibit high EQE of 6.5% and 4.8% with CIE (0.14, 0.13). The triplet energies (*E*_*T*_) estimated as 2.62 eV (DBTPIN) and 2.74 eV (NDBNPIN) are sufficient for the excitation of green and red phosphorescent dopants. Efficient green and red PhOLEDs with EQE of 23.0%/20.1% and 24.1%/21.3% have been harvested based on DBTPIN/NDBNPIN doped OLEDs,, respectively. White device with DBTPIN:Ir(MDQ)_2_(acac) (0.4%) exhibit maximum *η*_ex_ − 24.8%; *η*_c_ − 57.1 cdA^−1^; *η*_p_ − 64.8 lmW^−1^ with CIE (0.49, 0.40) than NDBNPIN:Ir(MDQ)_2_(acac) (0.4%) based device [*η*_ex_ − 23.1%; *η*_c_ − 54.6 cd A^−1^; *η*_p_ − 60.0 lm W^−1^, CIE (0.47, 0.42)]. We have reported structure modification strategy to harvest efficient full-color OLEDs by employing phenanthroimidazoles in doped, non-doped devices and red, green and white PHOLEDs.

## Experimental Section

### Synthesis of NDBNPIN and DPTPIN

#### 4′-bromo-N,N-diphenyl-[1,1′-biphenyl]-4-amine (TPAB-Br)

About 1.42 g of 1-bromo-4-iodobenzene (5 mmol), 2.89 g of (4-(diphenylamino) phenyl)boronic acid (10.0 mmol), 50 mg Pd(PPh_3_)_4_ (0.1 mmol) and 2 M K_2_CO_3_ (10 mL) was refluxed in toluene (30 mL) at 90 °C for 32 h (Scheme S1). The reaction mixture was treated with CH_2_Cl_2_ and dried, crude TPAB-Br was purified by column chromatography to afford white solid [CH_2_Cl_2_–petroleum ether (60–90 °C) (1:15)], yield: 84%. MS (EI): m/z 399.1, 401.3 (M^+^)^37^.

#### 4-naphthylcarbaldehyde-N,N-diphenyl-[1,1′-biphenyl]-4-amine (NCDBA)

The TPAB-Br (2 g, 5 mmol), 4-formylnaphthalen-1-yl-1-boronic acid (0.75 g, 5 mmol) and Pd(PPh_3_)_4_ (50 mg, 0.1 mmol) and 2 M K_2_CO_3_ (15 mL) was refluxed in toluene (30 mL) at 105 °C for 3 days with N_2_ stream. The reaction mixture was treated with CH_2_Cl_2_ and dried (Scheme S1).Yield 86.1%. ^1^H NMR (400 MHz, CDCl_3_): δ 6.45-6.68 (m, 8 H), 7.02 (t, 4 H), 7.25 (d, J = 8.42 Hz, 2 H), 7.41 − 7.68 (m, 7 H), 7.72 (d, J = 8.42 Hz, 2 H), 9.12 (d, J = 8.2 Hz, 1 H), 9.80 (s, 1 H); ^13^C NMR (400 MHz, CDCl_3_): δ 122.71, 123.68, 126.98, 128.32, 128.59, 129.74, 131.50, 132.86, 133.43, 135.47, 135.95, 136.89, 139.51, 141.12, 143.53, 191.52. MS (EI): m/z 475.23 (M^+^).

#### 4′-thienylcarbaldehyde-N,N-diphenyl-[1,1′-biphenyl]-4-amine (TCDBA)

The TPAB-Br (2 g, 5 mmol), 5-formylthiophen-2-yl-2-boronic acid (0.75 g, 5 mmol), Pd(PPh_3_)_4_ (50 mg, 0.1 mmol) and 2 M K_2_CO_3_ (15 mL) was refluxed in toluene (30 mL) (105 °C ;3 days;N_2_ stream). The reaction mixture was treated with CH_2_Cl_2_ and dried.Yield 85%. ^1^H NMR (400 MHz, CDCl_3_): δ 6.46-6.67 (m, 8 H), 7.15 (t, 5 H), 7.23 (d, J = 8.42 Hz, 2 H), 7.54 − 7.68 (m, 5 H), 9.68 (s, 1 H); ^13^C NMR (400 MHz, CDCl_3_): δ 122.68, 128.41, 129.54, 130.51, 132.74, 136.53, 138.72, 139.92, 141.09, 143.21, 148.32, 182.83. MS (EI): m/z 431.21 (M^+^).

#### 4-(2-(4-(4′-(diphenylamino)-[1,1′-biphenyl]-4-yl)naphthalen-1-yl)-1H-phenanthro [9,10-d]imidazol-1-yl)-1-naphthonitrile (NDBNPIN)

The NCDBA (4.5 mmol), 9,10-phenanthernequinone (5 mmol), 4-aminonaphthalene-1-carbonitrile (6 mmol) and ammonium acetate (61 mmol) was refluxed in ethanol (12 h; N_2_ atmosphere). The solution was extracted with dichloromethane and dried. Anal. Calcd. for C^60^H^38^N^4^: C, 88.43; H, 4.70; N, 6.87. Found: C, 88.36; H, 4.62; ^N, 6.75. 1^H NMR (400^ MHz, CDCl^_3_^): δ 6.43-6.52 (m, 6 H), 6.61 (d, J^ = ^8.0 Hz, 2 H), 7.01 (t,^ 4 H), 7.22 (d*, J* = *8.4 Hz*, 2 H), 7.32 (t, 2 H), 7.44 (d, J = 8.8 H*z*, 1 H), 7.54-7.61 (m, 10 H), 7.80–7.88 (m, 6 H), 8.12–8.20 (m, 3 H) 8.93 (d, J = 7.8 Hz, 2 H) (Fig. S2); ^13^C NMR (100 MHz, CDCl_3_): δ 109.88, 116.13, 122.12, 122.93, 123.11, 123.45, 123.97, 124.96, 126.13, 126.65, 127.53, 128.32, 128.47, 129.67, 130.15, 131.54, 133.21, 133.61, 133.89, 134.59, 135.47, 136.08, 139.99, 141.15, 149.51 (Fig. S3). MALDI-TOF MS: m/z 814.98, [M^+^]; calcd: 814. 31 (Fig. S6).

#### 4-(2-(5-(4′-(diphenylamino)-[1,1′-biphenyl]-4-yl)thiophen-2-yl)-1H-phenanthro[9,10-d] imidazol-1-yl)-1-naphthonitrile (DBTPIN)

The TCDBA (4.5 mmol), 9,10-phenanthernequinone (5 mmol), 4-aminonaphthalene-1-carbonitrile (6 mmol) and ammonium acetate (61 mmol) was refluxed in ethanol (12 h; N_2_ streame). The solution was extracted with dichloromethane and dried. Anal. Calcd. for C_54_H_34_N_4_S: C, 84.13; H, 4.45; N, 7.27. Found: C, 84.05; H, 4.37; N, 7.18. ^1^H NMR (400 MHz, CDCl_3_): δ 6.46–6.52 (m, 6 H), 7.0 (t, 6 H), 7.24 (d, J = 8 Hz, 2 H), 7.43 (d, J = 8.4 Hz, 1 H), 7.54 − 7.56 (t, 6 H), 7.80–7.88 (m, 8 H), 8.12–8.20 (m, 3 H), 8.96 (d, J = 7.6 Hz, 2 H) (Fig. S4); ^13^C NMR (100 MHz, CDCl_3_): δ 109.87, 116.15, 121.69, 122.45, 122.68, 123.35, 125.64, 126.79, 128.84, 129.75, 130.54, 131.58, 132.98, 134.10, 136.51, 138.32, 139.51, 141.06, 141.75, 143.54 (Fig. S5). MALDI-TOF MS: m/z 771.01, [M^+^]; calcd: 770. 25 (Fig. S6).

### Devices fabrication

ITO glass (resistance 20 Ω/sq) was cleaned with acetone, isopropanol and deionised water and dried (120 °C) followed by UV-zone treatment (20 min). Fabrication was made by deposition method in a vacuum (4 × 10^−5^ mbar). Organic materials and metal electrodes are evaporated at a rate of 0.4 Å s^−1^ for 1–4 Å s^−1^, respectively. Thickness of each layer was measured with quartz crystal thickness monitor. The EL spectra/CIE coordinates were measured with USB-650-VIS-NIR spectrometer (Ocean Opitics, Inc, USA). Current density-voltage-luminance (J-V-L) were measured by computer-controlled source meter (Keithley 2450) equipped with light intensity meter LS-110 under ambient atmosphere. The EQEs were determined from luminance, current density and EL spectrum.

## Single carrier device

Hole-only:(I) ITO/HATCN (8 nm)/DBTPIN or NDBNPIN (50 nm)/HATCN (8 nm)/LiF (1 nm)/Al (100 nm);(II) ITO/HATCN (8 nm)/DBTPIN (30 nm): 5 wt % Ir(ppy)_3_ or NDBNPIN (30 nm):5wt % Ir(ppy)_3_/HATCN (8 nm)/LiF (1 nm)/Al (100 nm); (III) ITO/HATCN (8 nm)/ CBP (30 nm):5 wt % Ir(ppy)_3_ or CBP (30 nm): 5 wt % Ir(MDQ)_2_ (acac)/HATCN (8 nm)/LiF (1 nm)/Al(100 nm) and electron-only:(IV)ITO/TPBi (8 nm)/DBTPIN or NDBNPIN (50 nm)/TPBi (8 nm)/LiF(1 nm)/Al (100 nm);(V) ITO/TPBi (8 nm)/DBTPIN (30 nm): 5 wt % Ir(ppy)_3_ or NDBNPIN (30 nm):5 wt % Ir(ppy)_3_/TPBi (8 nm)/LiF (1 nm)/Al (100 nm);(VI) ITO/TPBi (8 nm)/CBP (30 nm): 5 wt % Ir(ppy)_3_ or CBP (30 nm): 5 wt % Ir(MDQ)_2_ (acac)/TPBi (8 nm)/LiF (1 nm)/Al (100 nm) were made (Fig. 6; Tables S1–S3)^38–67^.

## Measurement

^1^H and ^13^C NMR and mass spectra were recorded at 298 K on Bruker 400 MHz spectrometer and Agilent (LCMS VL SD), respectively. Absorption (solution and film) were recorded on Perkin-Elmer Lambda 35 and Lambda 35 spectrophotometer with integrated sphere (RSA-PE-20), respectively. PerkinElmer LS55 fluorescence spectrometer and fluorescence spectrometer Model-F7100 with integrating sphere was employed to analyse PL and absolute quantum yield, respectively. Thermogravimetric analysis (TGA) and differential scanning calorimetric (DSC) were recorded with PerkinElmer thermal analysis system and NETZSCH-DSC-204, respectively (10 °C min^−1^; N_2_ flow rate of 100 mL min^−1^). Lifetime was estimated with time correlated single-photon counting (TCSPC) method on Horiba Fluorocube-01-NL lifetime system. Cyclic voltammetry was performed with potentiostate CHI 630 A electrochemical analyzer. The HOMO [*E*_*HOMO*_ = −(*E*_*ox*_ + 4.8 eV)] energies and LUMO [*E*_*LUMO*_ = (*E*_*red*_ − 4.8 eV)] energies were calculated using oxidation and reduction potentials, respectively.

## Computational Details

The ground and excited state analysis were studied by using Gaussian 09 program^68^ and multifunctional wavefunction analyzer (Multiwfn)^69^ (Figs. S7–S14).

## Supplementary information

Supplementary information.

## Data Availability

The authors declare that data in our manuscript are available.
