# Supplementary material for: A novel hot exciton blue fluorophores and white organic light-emitting diodes with simplified configuration
Source: Sci Rep. 2020 Mar 20;10:5114. doi: 10.1038/s41598-020-62029-4 (PMC7083847; doi:10.1038/s41598-020-62029-4)
Supplement: Supplementary file 1 — Supplementary information. [file 41598_2020_62029_MOESM1_ESM.docx]

**Supplementary Information**

**A novel hot exciton blue fluorophores and white organic light-emitting diodes with simplified configuration**

**Jayaraman Jayabharathi*, Sekar Panimozhi, Venugopal Thanikachalam**

*Department of Chemistry, Annamalai University, Annamalainagar 608 002, Tamilnadu, India*

* Tel: +91 9443940735; *E-mail address:* [jtchalam2005@yahoo.co.in](mailto:jtchalam2005@yahoo.co.in).

Address for correspondence

Dr. J. Jayabharathi

Professor of Chemistry

Department of Chemistry

Annamalai University

Annamalai nagar 608 002

Tamilnadu, India.

Tel: +91 9443940735

E-mail: [jtchalam2005@yahoo.co.in](mailto:jtchalam2005@yahoo.co.in)

**Supplementary Information Contents:**

**SI-I: Scheme S1**

**SI-II: Solvatochromism for HLCT character**

**SI-III: Figures S1-S8**

**SI-IV: Potential energy scan (PES)**

**SI-V: Figures S9-S14**

**SI-VI: Tables S1-S3**

**SI-I: Scheme S1**. Synthetic route of NDBNPIN and DBTPIN.


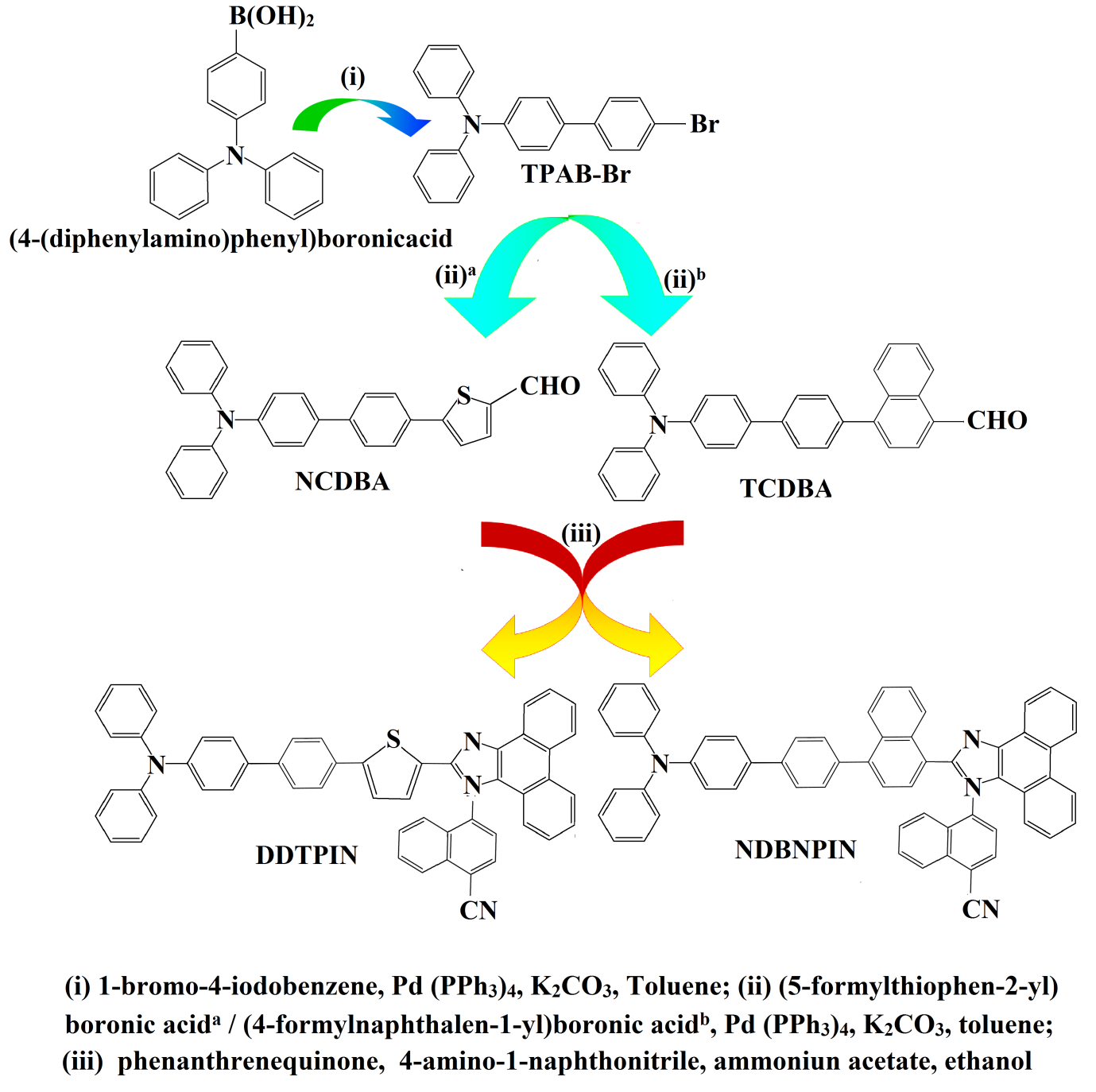


**SI-II: Solvatochromism for HLCT character**

The solvatochromic effect using Lippert-Mataga plot has been displayed in Fig. S1. When solvent polarity increased the blue emitters exhibit a larger red shift which supports the charge transfer (CT) in these molecules. From Lippert-Mataga plot, the ground state dipolemoment (µ_g_) can be calculated: hc(ῦ_abs -_ ῦ_flu_) = hc(${hcῦ}_{\mathrm{abs}}^{\nu ac}$ _-_${hcῦ}_{flu}^{\nu ac}$)  _+_ 2 (µ_e_ - µ_g_)^2^ /$a_{o}^{3}$ [(ε – 1/ 2 ε + 1) – ½ (n^2^ -1/ 2n^2^ +1)] [*µ_g_* and *µ_e_* - ground state and excited state dipolemoment, *ῦ_abs_* and $ῦ_{\mathrm{abs}}^{\nu ac}$ - solvent-equilibrated absorption maxima (λ_abs_) and extrapolated to gas phase, *ῦ_flu_* and $ῦ_{flu}^{\nu ac}$ - solvent equilibrated fluorescence maxima (λ_emi_) and extrapolated to gas-phase, respectively, *a*_o_ - onsager cavity and *ε* and *n* solvent dielectric constant and refractive index, respectively]. The non-linear correlation of Stokes shift *Vs* solvent polarity function reveal that there is transformation of fitted line between ethyl ether and methylene chloride: non-linear correlation supports the presence of both locally excited state (LE) and charge transfer excited state (CT). The large *μ_e_* in high polar medium is in close with *μ_e_* of charge-transfer molecule, 4-(*N*, *N*-dimethylamino) benzonitrile (23.0 D). All these results show that CT dominates in more polar medium and LE dominates in low polar solvent and there is mixed contribution of LE and CT in medium polar solvents.

The optical characteristics of emissive materials were studied in solution as well as solid by absorption and emission studies. When cyano group is incorporated, the modification in charge-transfer state red-shifted the absorption and emission. The strong absorption at ~ 276 nm is due to π-π* transition originates from phenyl ring to imidazole ring. The absorption at 382 nm (DBTPIN) and 378 nm (NDBNPIN) is attributed to intramolecular CT from donor to acceptor unit.^1^ New born emitters exhibit similar absorption (sol/film) 296, 382(ε_max_ =26178 cm^-1^ M^-1^)**/**246, 426 (NDBNPIN) and 276, 378 (ε_max_ = 26455cm^-1^ M^-1^)**/**318, 418 nm (DBTPIN), since the core fragment is not different.^2-4^ The emission of DBTPIN and NDBNPIN is observed at 440 and 428 nm in solid (FWHM**~**28 nm), the red-shift is unlikely due to aggregation but may be because of excited state configurational change. The CN group rarely influences the electronic properties as well as band-gap. It is in accordance with quantum chemical calculations. The emission peak is red shifted as increasing the solvent polarity (Fig. S1) because of polarization induced optical shift. The PL spectra gradually widened peaks indicates that their excited state have strong CT character when compared to ground state and further stabilized by polar solvents. Compared to NDBNPIN, DBTPIN shows blue shift of absorption as well as emission due to the presence of thienyl fragment. The increased % LE along with decreased % CT in S_1_ HLCT is probably the reason for the observed blue shift. The FWHM of absorption peak of DBTPIN (33 nm) is reduced compared to NDBNPIN (45 nm). This result shows decrease of % CT of DBTPIN in S_1_ which is in accordance with NTO analysis (S_0_→S_1_ transition).^5^ The DBTPIN shows solvatochromic smaller red shift in emission (28 nm) compared to that of NDBNPIN (64 nm) (Fig. S1). In the same way a small absorption red-shift *viz.,* 18 nm and 26 nm is observed for DBTPIN and NDBNPIN (Fig. S1).

**SI-III: Figures S1-S8**

**Figure S1.** (a) Lippert–Mataga plot; solvatochromic b) absorption and c) emission spectra of NDBNPIN and DBTPIN.


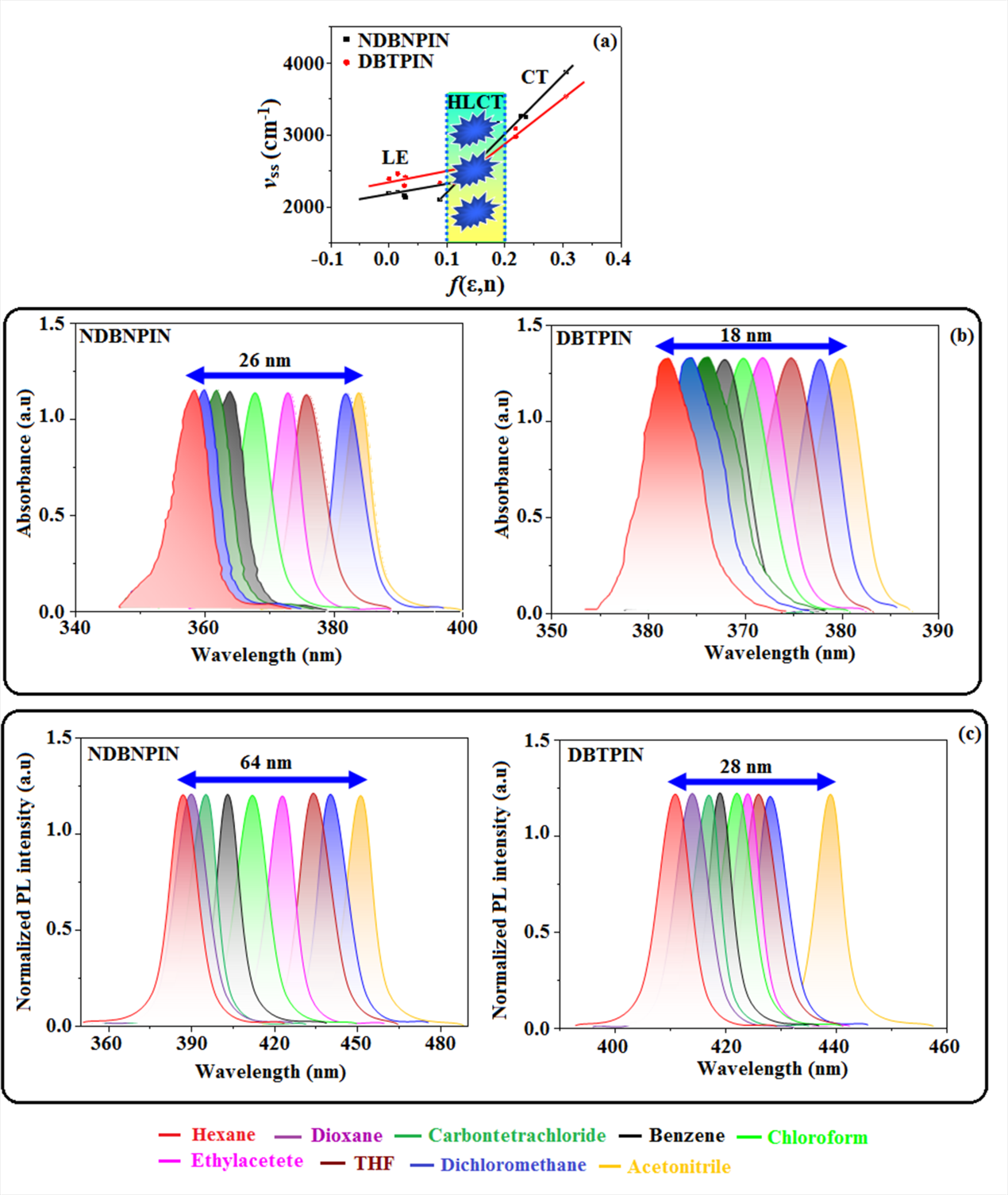


**Figure S2.** ^1^H NMR spectrum of 4-(2-(4-(4'-(diphenylamino)-[1,1'-biphenyl]-4-yl)naphthalen-1-yl)-1H-phenanthro [9,10-d]imidazol-1-yl)-1-naphthonitrile (NDBNPIN)


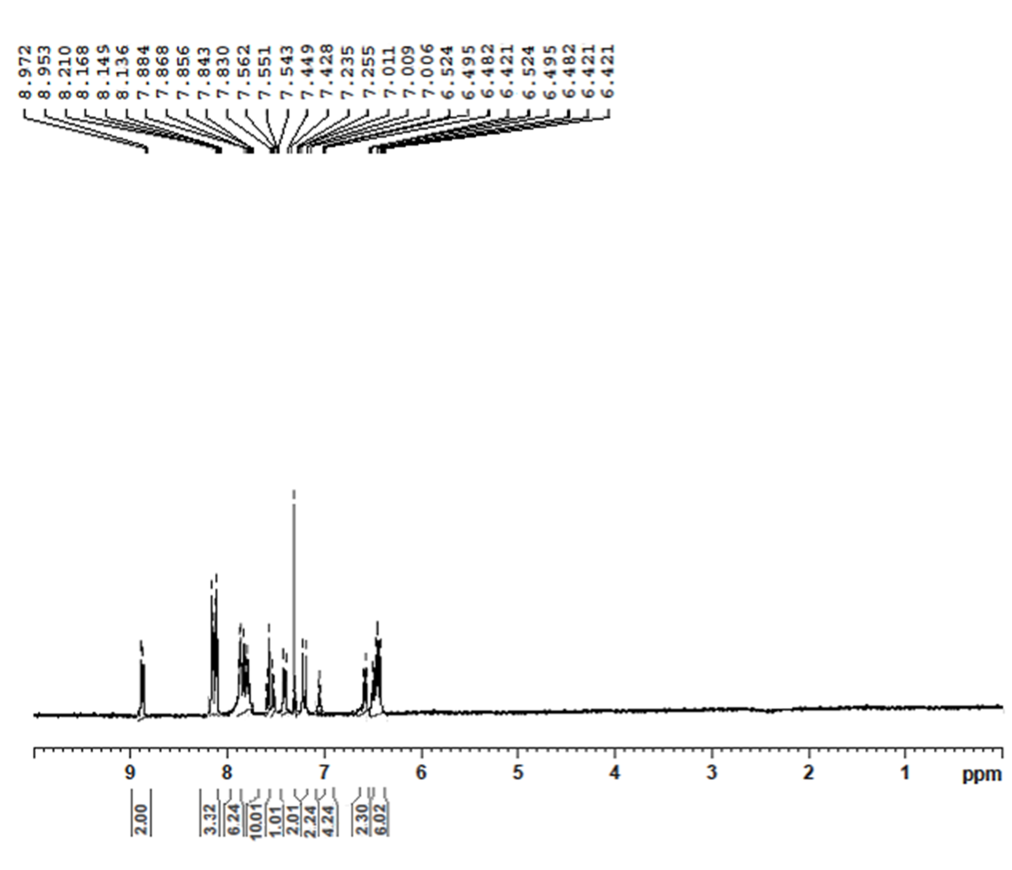


**Figure S3.** ^13^C NMR spectrum of 4-(2-(4-(4'-(diphenylamino)-[1,1'-biphenyl]-4-yl)naphthalen-1-yl)-1H-phenanthro [9,10-d]imidazol-1-yl)-1-naphthonitrile (NDBNPIN)


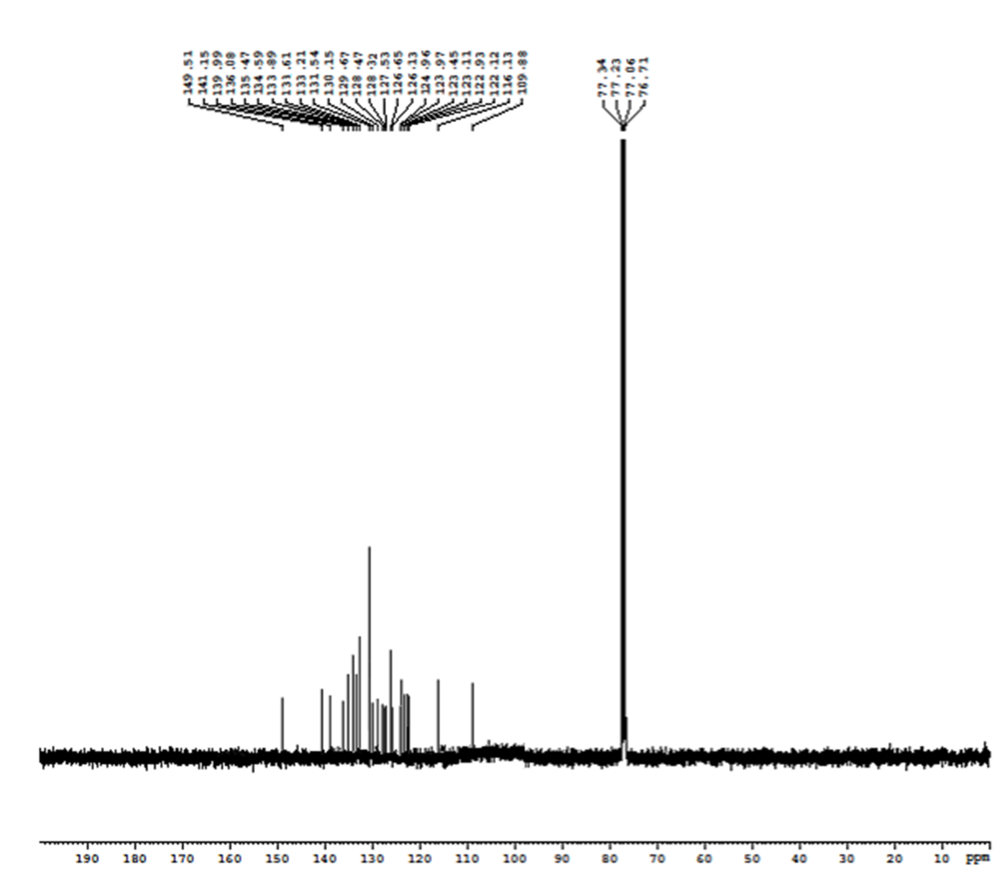


**Figure S4.** ^1^H NMR spectrum of 4-(2-(5-(4'-(diphenylamino)-[1,1'-biphenyl]-4-yl)thiophen-2-yl)-1H-phenanthro[9,10-d] imidazol-1-yl)-1-naphthonitrile (DBTPIN)


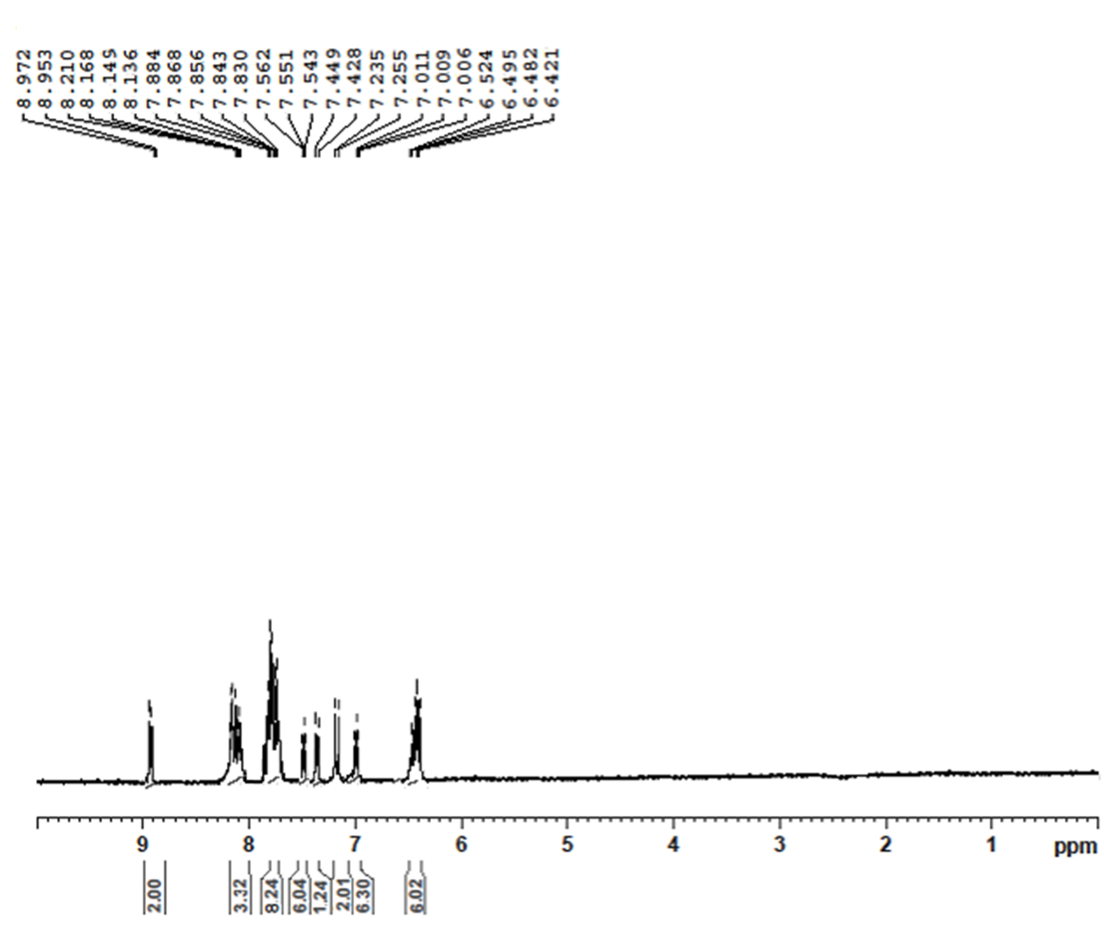


**Figure S5.** ^13^C NMR spectrum of 4-(2-(5-(4'-(diphenylamino)-[1,1'-biphenyl]-4-yl)thiophen-2-yl)-1H-phenanthro[9,10-d] imidazol-1-yl)-1-naphthonitrile (DBTPIN)


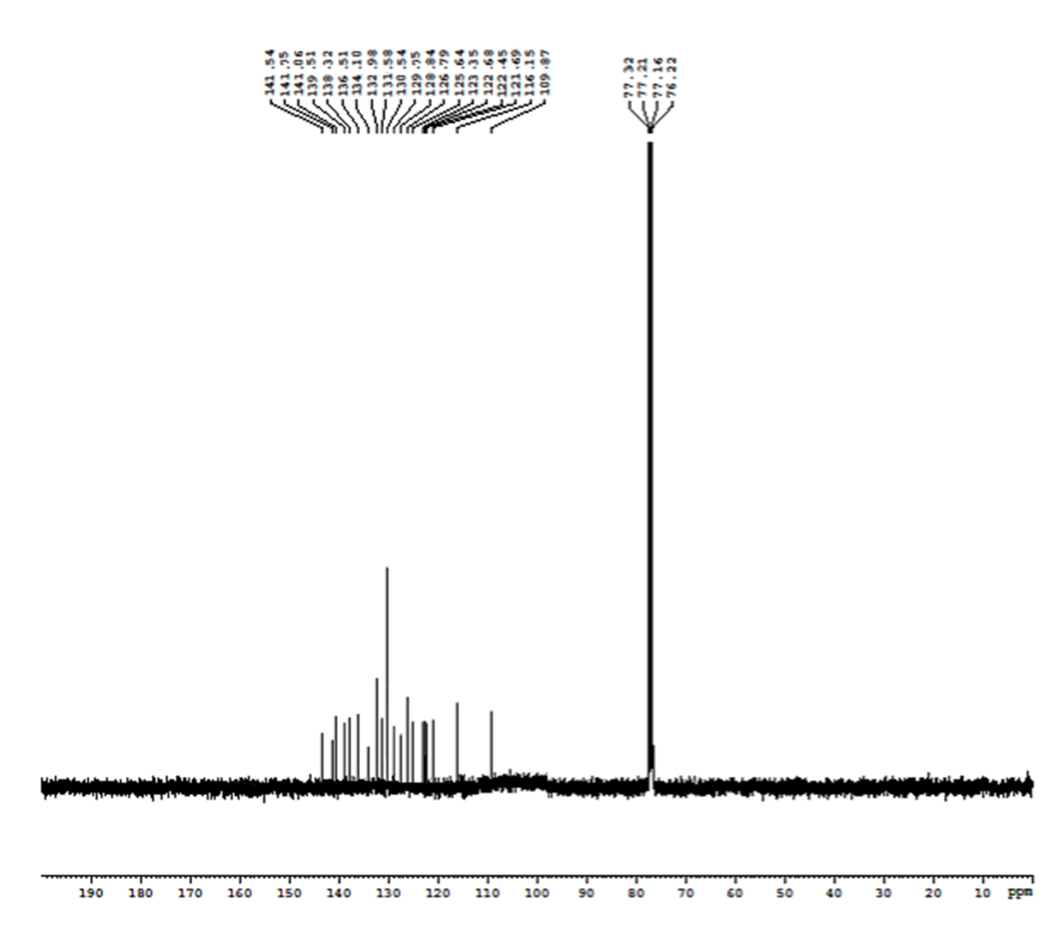


**Figure S6.** MALDI-TOF mass spectra of (a) NDBNPIN and (b) DBTPIN
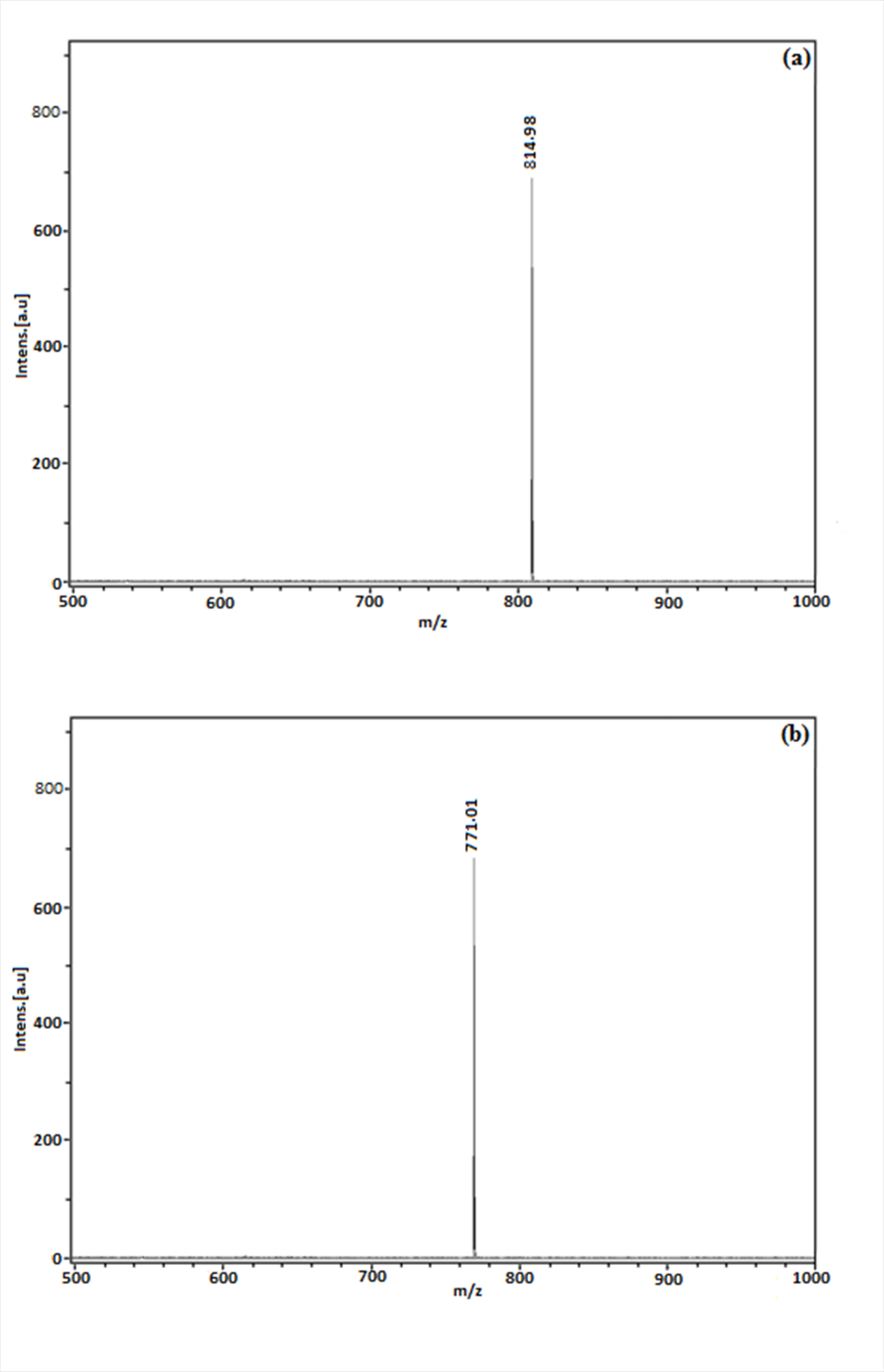


Solvatochromic shifts^6,7^ confirmed that low-lying S_1_ excited state must possesses CT character. The CT % in S1 state of DBTPIN is less than NDBNPIN whereas LE % of DBTPIN is larger than NDBNPIN. In S_0_→S_1_ and S_0_→S_2_ transitions, the HLCT was composed of CT state followed by LE state and exhibit larger oscillator strength [*f_S0–S1_* / *f_S0–S2_* = 0.012/0.062 (NDBNPIN: Fig. S7) 0.667/0.489 (DBTPIN: Fig. S8)] compared with other S_0_-S_n_ transitions. This is because of larger LE % in HLCT which is necessary for higher efficiency OLEDs. The hole as well as particle of S_0_→S_1_ and S_0_→S_2_ transitions in NDBNPIN and DBTPIN consist of two different configurations with comparable contributions: C_2_ direction takes up one and N_1_ direction takes up other: LE and HLCT states have one squint each. This implies that these compounds show a well mixed CT and LE. In D-ᴨ-A molecules, CT and LE components are mutually perpendicular in HLCT.

The twist angles (θ_2_ and θ_3_) in D-π-A linkage of NDBNPIN and DBTPIN could be the origin for CT and LE intercross: S_1_ state remained unchanged with increasing solvent polarity whereas the S_3_ state decreased to intercross with S_1_ state at moderate polarity and to be much lower than S_1_ state at high polarity (Fig. S1). This reveals the HLCT as single emissive state. The LE and CT states of emissive materials NDBNPIN and DBTPIN show non-uniform properties in different polar solvents because of different excited-state dipole moments. As the polarity increases, CT state is stabilized due to strong interaction of the solvent field with CT excited state (large dipole moment) and LE remains unchanged however, in low-polarity solvents, the LE being stabilized (low-lying excited state).

This transition among the excited states suppresses non-radiative decay, increasing both the efficiency of photoluminescence and oscillator strength. This is supported by optical studies.

**Figure S7.** Natural transition orbital pairs with (HONTOs and LUNTOs) transition character analysis for singlet states (S_1_-S_5_) of NDBNPIN [*f*-oscillator strength and % weights of hole-particle].

**
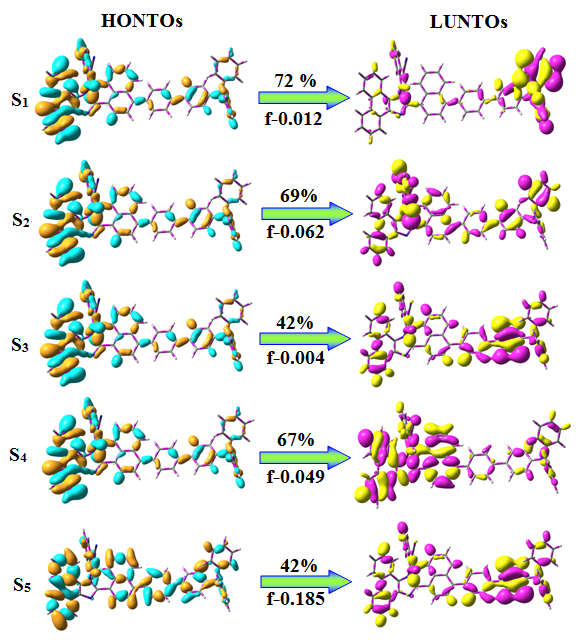
**

**Figure S8.** Natural transition orbital pairs with (HONTOs and LUNTOs) transition character analysis for singlet states (S_1_-S_5_) of DBTPIN [*f*-oscillator strength and % weights of hole-particle].

**
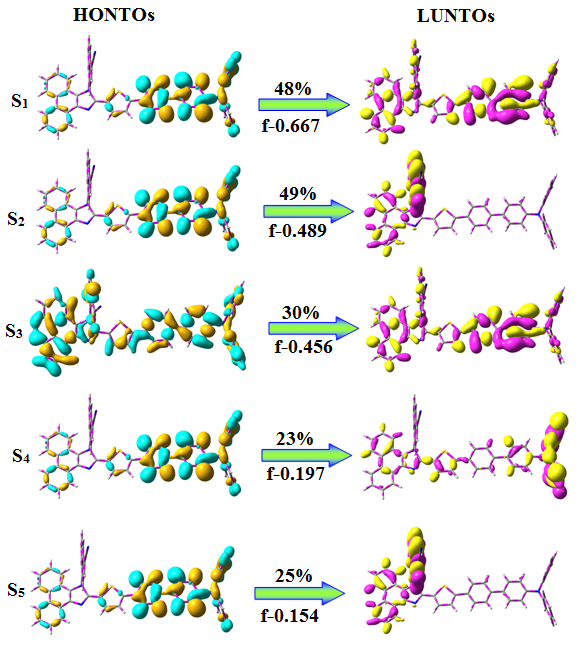
**

**SI-IV: Potential energy scan (PES)**

The optimization of ground (S_0_) as well as excited (S_1_) geometries of D-ᴨ-A compounds were made using Gaussian-09 (Fig. S9). The ground state energy results by potential energy scan reveal that **θ_1_**: DBTPIN -120.0°-123.2°: NDBNPIN -119.0°-121.6°; **θ_2_:** DBTPIN -119.0°-120.1°: NDBNPIN -121.0°-124.1°; **θ_3_:** DBTPIN -112.6°-114.2°: NDBNPIN -119.0°-120.8 (Fig. S10).

**SI-V: Figures S9-S14**

**Figure S9.** Molecular structure, ground and excited state geometries with dihedral angles of NDBNPIN and DBTPIN.

**
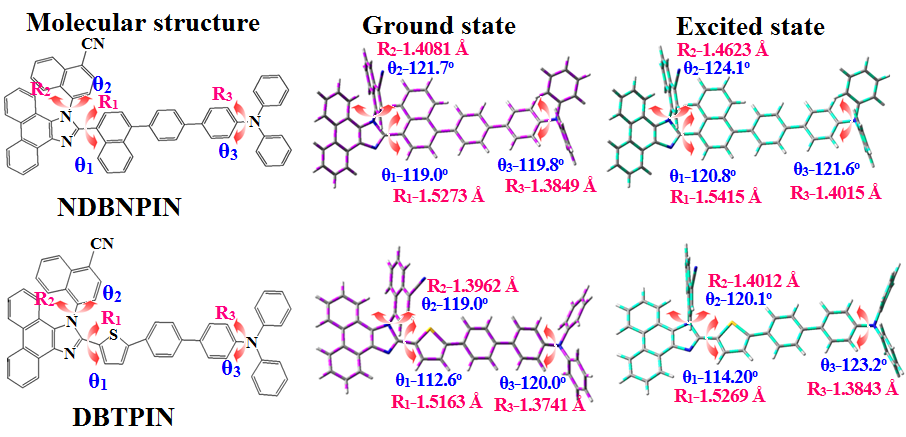
**

**Figure S10.** Potential energy scan of (a) NDBNPIN and (b) DBTPIN; potential energy scan of excited state of NDBNPIN and DBTPIN (c&d) with increasing solvent polarity.


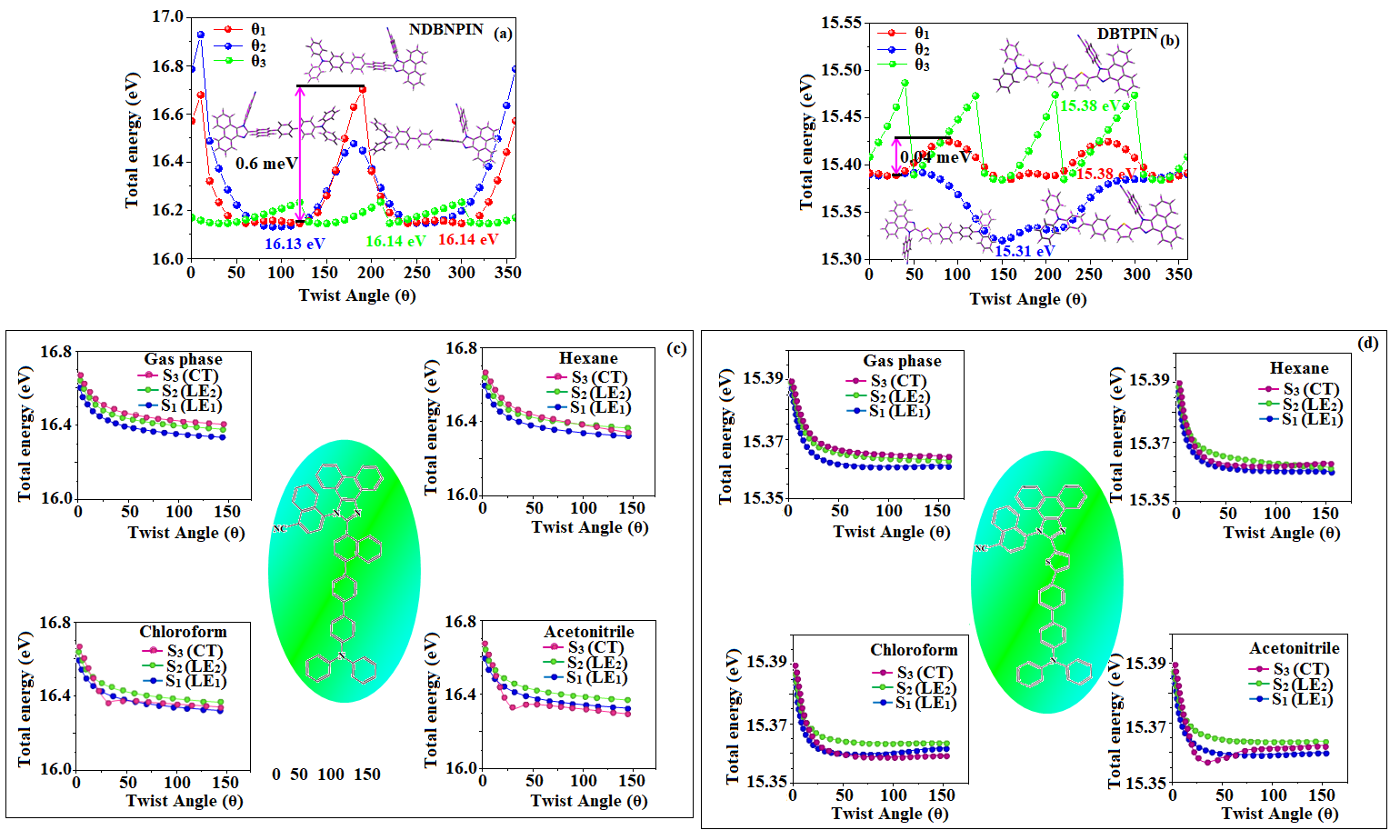


The incorporation of cyanonaphthyl group in phenanthrimidazole unit increases the charge transfer state, as a result, efficient reverse intersystem crossing process would be facilitated to enhance the fraction of singlet generation.^8^ The thienyl-phenyl (DBTPIN) and naphthyl-phenyl bridge (NDBNPIN) with phenanthrimidazole plane in the backbone enhanced the horizontal locally excited state to balance the CT component resulting in high photoluminescence quantum yield (Φ_s/f_).^9^ Clipping the linkage between the triphenylamine and phenanthrimidazole plane by thienyl phenyl or naphthyl phenyl is beneficial to suppress the aggregation effect to maintain the color purity of OLEDs. In D-ᴨ-A materials ground state (S_o_) geometry, naphthonitrile part (θ_1_) as well as thienyl (DBTPIN) or naphthyl (NDBNPIN) moiety (θ_2_) are largely twisted over phenanthrimidazole.

The large twist-angles of rigid DBTPIN and NDBNPIN is because of strong repulsive interaction of the hydrogens of TPA with hydrogens of remaining molecule. The twist angle (θ_2_) of excited DBTPIN and NDBNPIN are increased (120.1° and 124.1°) on comparison to the twist angle of S_0_. The same way, excited state twist angle (θ_1_) was small for DBTPIN (114.2°) and NDBNPIN (120.8°). The DBTPIN and NDBNPIN bond length (R1) are increased (0.01 and 0.07 Å, respectively) on moving from ground (S_0_) to excited state (S_1_) on switching from ground (S_0_) to excited (S_0_) state geometry; small structural modification of DBTPIN increases the photoluminance efficiency (*η_PL_*). These orthogonal dihedral angles confirmed the non-coplanar twisting conformation of DBTPIN and NDBNPIN which suppresses the red shift and harvested high *η*_ex_ in film by restraining intermolecular interaction.^10-12^

The electronic transitions of NDBNPIN and DBTPIN were evaluated for S_1_-S_10_ states for ground and excited states using Gaussian B3LYP/6-31G (d, p) and TD-PBEPBE/6-31G (d, p) (Fig. S7&S8). The formation of single emissive state can be analyzed through excitation energies of LE and CT states. Similar hole-electron wave function between S_1_ and S_2_ is observed in both NDBNPIN, DBTPIN indicates a quasi-equivalent hybridization between LE and CT states, as a result of their almost isoenergies of initial LE and CT states (Fig. S11).

**Figure S11.** (a) Energy level diagram of singlet (S) and triplet (T) and (b) schematic diagram of hybridization processes of LE and CT states of NDBNPIN and DBTPIN.


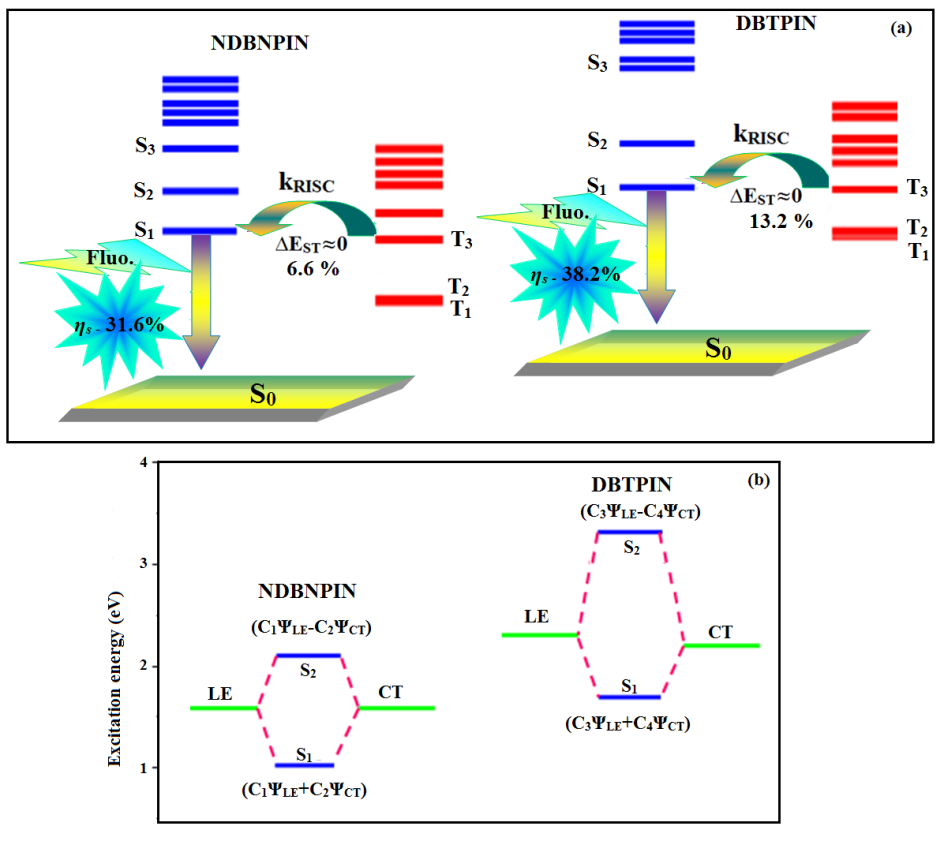


Therefore, degree of hybridization between LE and CT states depend not only initial *E_LE_-E_CT_* energy gap but also their interstate coupling strength. Compared with non-equivalent hybridization, quasi-equivalent hybridization is expected to achieve the combination of high *η_PL_* and high *η_s_* to maximize EL efficiency of fluorescent OLED materials due to more balanced LE and CT components in HLCT state of NDBNPIN and DBTPIN. In NDBNPIN, DBTPIN the LE state is stabilized than CT state and energy gap (*E_S2_-E_S1_*) is small when compared with their parent compounds results quasi hybridization which improves OLED efficiency. Composition of HLCT can be discussed from wave function of electron–hole pairs transition density matrix (TDM) and plot them in two-dimension color-filled map (Fig. S12-NDBNPIN Fig. S13 -DBTPIN).

**Figure S12**.(a) Transition density matrices (TDM) of NDBNPIN for [S_1_–S_3_ states] and (b) hole and particle distribution [S_1_–S_3_ states: green-increasing electron density and blue-decreasing electron density]


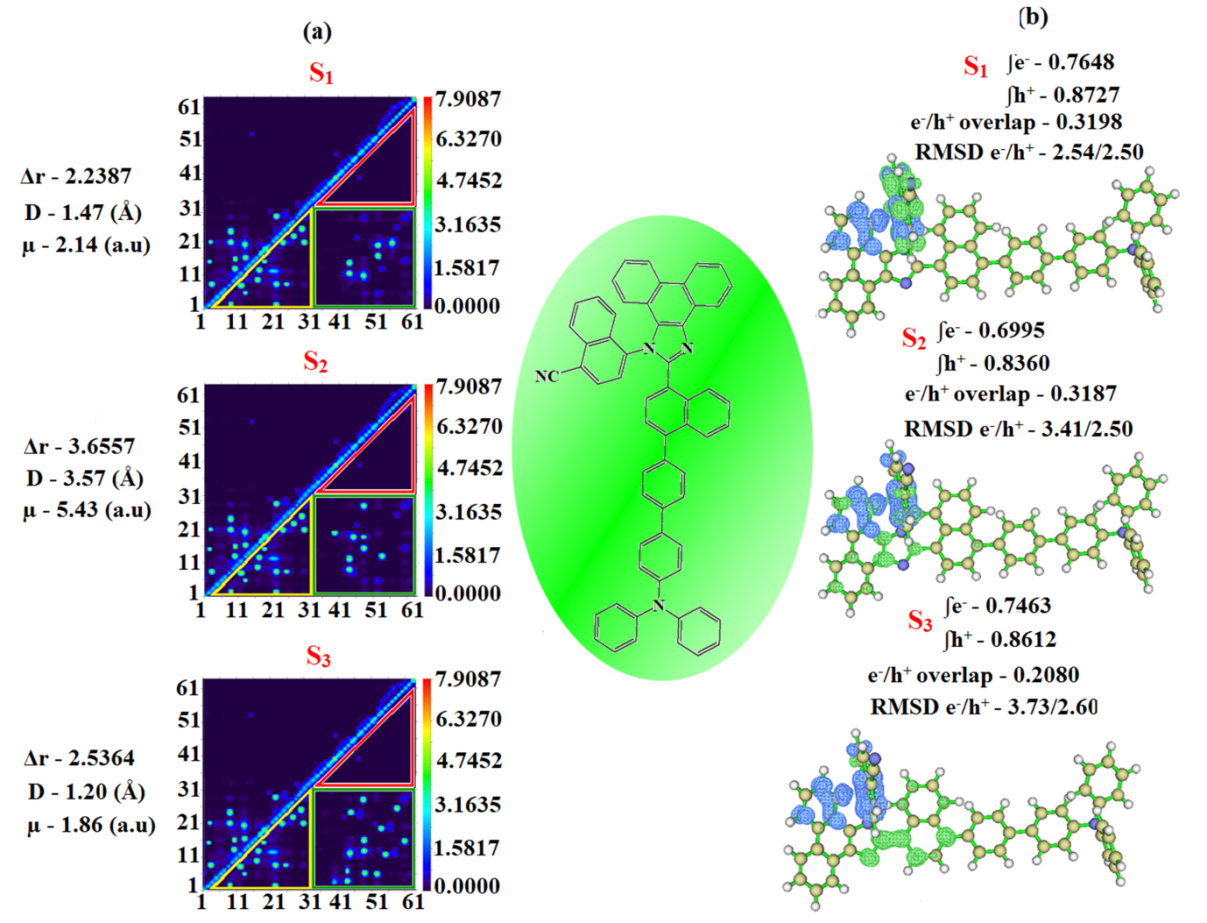


**Figure S13.** (a) Transition density matrices (TDM) of DBTPIN for [S_1_–S_3_ states] and (b) hole and particle distribution [S_1_–S_3_ states: green-increasing electron density and blue-decreasing electron density]

**
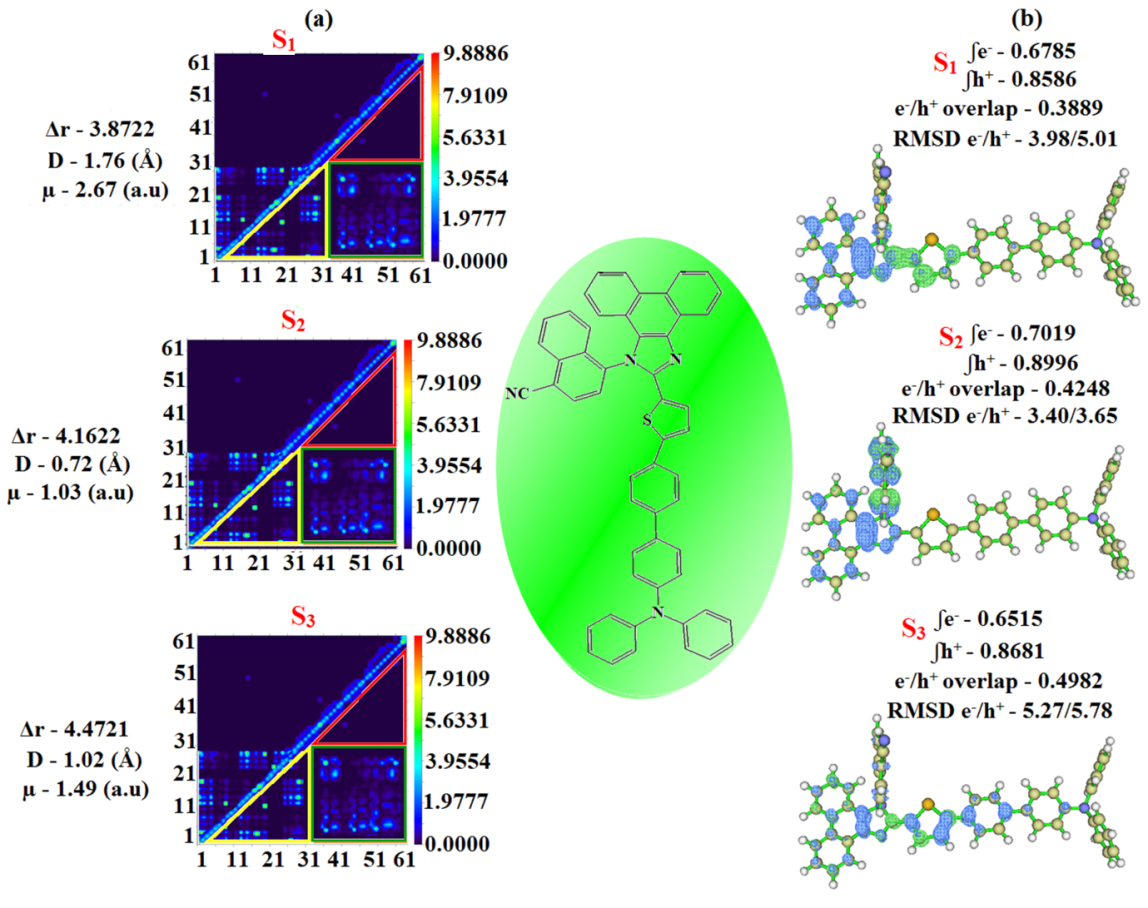
**

The diagonal region represents the LE component localized on the main backbone, while off–diagonal region shows CT component. This also supports that HLCT state also contributes to hybridization apart from LE and CT states. Upon excitation, electron is transferred from donor and localized on acceptor. Depending upon intramolecular geometrical and electronic coupling, transferred electron is delocalized from the region of nearby donor molecule to the vicinity of acceptor. This effect can be qualitatively studied by analyzing electron density distribution at ground and excited states.

The *D_CT_* of DBTPIN and NDBNPIN was calculated to be 0.592 and 0.691, respectively (Fig. S14). For both DBTPIN and NDBNPIN, the non-zero *t* is negative in all directions: overlap of hole and electron is very severe and eign value is greater than 0.96, supports the hybridization and described in terms of dominant excitation pair in terms of 94 % of transition.

**Figure S14.** Computed difference in total density for ground and excited states [Δρ (r)=ρEx (r)-ρGs (r); isosurface for NDBNPIN, DBTPIN, (0.0000006 a.u) and graphical representation of DCT and centroid of charges [C+(r) / C-(r); isosurface for NDBNPIN (0. 29 a.u) and for DBTPIN (0.1 a.u)]


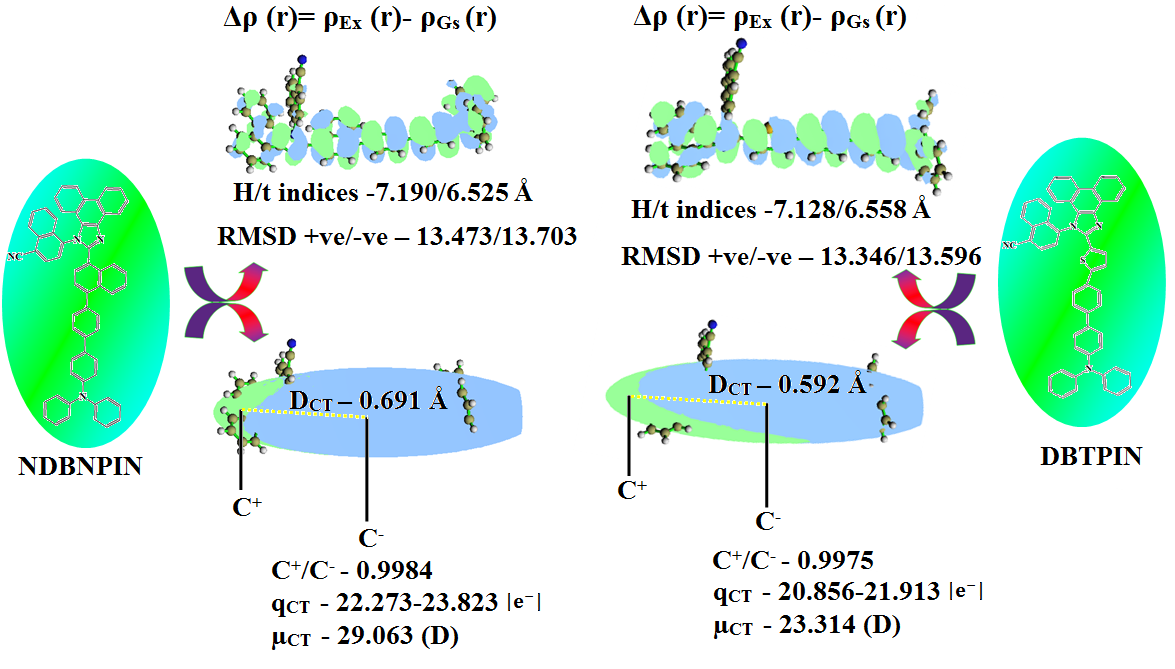


The Δr intex (equation S1) is average hole (h^+^)–electron (e^-^) distance (d_h+-e-_) upon excitation which tells the nature of the excitation type, LE or CT: valence excitation (LE) is related to short distances (d_h+-e-_) while larger distances (d_h+-e_) related to CT excitation. The triplet exciton is transformed to the singlet excitons in DBTPIN and NDBNPIN *via* RISC process with high energy excited state (hot CT channel) which is beneficial for triplet exciton conversion in electroluminescence process without any delayed fluorescence. The CT excitons are formed with weak binding energy (*E_b_*) on higher excited states. As a result, the exciton utilization (η_s_) can be harvested in DBTPIN and NDBNPIN like phosphorescent materials. The quasi-equivalent hybridized material DBTPIN and NDBNPIN exhibits excellent device performances due to fine modulation in excited states: enhanced LE component and hybridization between LE and CT components results high *η_PL_* and high *η_s_*. The coexisting LE/CT composition in DBTPIN and NDBNPIN harvested high *η_PL_* and high *η_s_* and enhanced the OLEDs performances.

**SI-VI: Tables S1-S3**

**Table S1**: Summary of device efficiencies with reported non-doped emitters.

| **Emitter** | **V_on_(V)** | **L(cd/m^2^)** | **EL(nm)** | **ƞ_c_(cd/A)** | **ƞ_p_(lm/W)** | **CIE(x,y)** | **ref** |
| --- | --- | --- | --- | --- | --- | --- | --- |
| **NDBNPIN** | **2.5** | **-** | **441** | **5.8** | **6.1** | **0.14,0.13** | **This work** |
| **DBTPIN** | **2.3** | **-** | **427** | **7.0** | **7.4** | **0.14,0.13** | **This work** |
| Cz-DPVI | 3.4 | - | 419 | 4.9 | 4.3 | (0.15,0.08) | 38 |
| PPI | 3.8 | - | 412 | 0.71 | 0.40 | (0.161,0.065) | 39 |
| mTPA-PPI | 3.2 | 4065 | 404 | 0.84 | 0.48 | (0.161,0.049) | 39 |
| L-BPPI(50nm) | 8.5 | 70 | 440 | 0.01 | - | (0.16,0.11) | 40 |
| L-BPPI(40nm) | 6.5 | 295 | 440 | 0.13 | - | (0.16,0.11) | 40 |
| L-BPPI(30nm) | 5.0 | 420 | 440 | 0.40 | - | (0.16,0.10) | 40 |
| L-BPPI(20nm) | 4.5 | 391 | 440 | 0.68 | - | (0.16,0.10) | 40 |
| Z-BPPI(50nm) | 6.5 | 105 | 440 | 0.07 | - | (0.17,0.12) | 40 |
| Z-BPPI(40nm) | 5.0 | 502 | 440 | 0.34 | - | (0.16,0.12) | 40 |
| Z-BPPI(30nm) | 4.5 | 267 | 440 | 0.45 | - | (0.16,0.12) | 40 |
| Z-BPPI(20nm) | 5.0 | 100 | 440 | 0.31 | - | (0.16,0.11) | 40 |
| MADN (BUBD) | 7.8 | - | 440 | 2.1 | - | (0.15,0.10) | 41 |
| CPPPI | - | 3322 | 420 | 0.65 | 0.48 | (0.165,0.050) | 42 |
| PPICPPPI | - | 4329 | 428 | 1.53 | 0.86 | (0.166,0.056) | 42 |
| PhBPI | 2.8 | - | 450 | 1.87 | 1.85 | - | 43 |
| bilayer-TPBI | 3.2 | - | 468 | 2.03 | 1.00 | (0.15,0.15) | 44 |
| TPA-BPI | 2.8 | - | 448 | 1.83 | 1.58 | (0.15,0.09) | 45 |
| DPVBi | 7.5 | - | 457 | 0.03 | - | (0.15, 0.13) | 46 |
| 3,6-DPVTCz | 5.0 | - | 449 | 0.11 | - | (0.15, 0.11) | 46 |
| PEDOt-PSS: 3 (100nm) | 4.0 | 2800 | 460 | 0.61 | 0.14 | (0.15,0.14) | 47 |
| PEDOt-PSS :3(50 nm) | 3 | 10600 | 407 | 1.68 | 1.10 | (0.16,0.13) | 47 |
| PEDOt-PSS :4 (40 nm) | 2.5 | 21200 | 392 | 1.90 | 1.55 | (0.16,0.14) | 47 |
| TTP-TPI | 3.1 | - | - | 2.10 | 1.88 | 0.16, 0.05 | 47 |
| DPT-TPI | 2.9 | - | - | 3.13 | 3.22 | 0.16, 0.07 | 47 |
| PMSO | 3.2 | - | - | 4.64 | 4.0 | 0.152, 0.077 | 48 |
| PPI-2TPA | 3.0 | - | - | 4.40 | 4.60 | 0.150, 0.063 | 49 |
| PPI-2NPA | 3.0 | - | - | 3.98 | 3.88 | 0.151, 0.066 | 50 |
| TPIBNCz | 3.2 | - | - | 3.29 | 2.80 | 0.157, 0.074 | 51 |
| PPI-PID + CBP | 3.15 | - | - | 4.13 | - | 0.151, 0.076 | 52 |

**Table S2:** Summary of device efficiencies with reported green emitters.

| **Emitter** | **V_on_(V)** | **L(cd/m^2^)** | **EL(nm)** | **ƞ_c_(cd/A)** | **ƞ_p_(lm/W)** | **CIE(x,y)** | **ref** |
| --- | --- | --- | --- | --- | --- | --- | --- |
| **NDBNPIN:Ir(ppy)_3_** | **2.7** | **-** | **512** | **27.1** | **31.2** | **0.31,0.60** | **This work** |
| **DBTPIN:Ir(ppy)_3_** | **2.6** | **-** | **509** | **29.0** | **35.4** | **0.31,0.60** | **This work** |
| TPPI | 4.2 | - | - | - | - | - | 53 |
| CN-BPPI | 5.6 | - | - | 0.78 | 0.26 | 0.17,0.22 | 54 |
| phImTD | 3.6 | - | - | 1.34 | 0.82 | 0.151, 0.098 | 55 |
| phImPOTD | 3.6 | - | - | 1.13 | 0.50 | 0.151,0.084 | 56 |
| SiBPI | 3.8 | - | - | 1.04 | - | 0.166, 0.046 | 57 |
| DPACTPI | 3.6 | - | - | 1.84 | 1.75 | 0.165, 0.068 | 58 |
| DPACPhTPI | 3.3 | - | - | 1.84 | 4.02 | 0.160, 0.078 | 59 |
| PITO | 3.2 | - | - | 7.17 | - | 0.17, 0.31 | 59 |
| PISF | 3.2 | - | - | 1.96 | - | 0.16, 0.09 | 59 |

**Table S3:** Summary of device efficiencies with reported red emitters.

| **Emitter** | **V_on_(V)** | **L(cd/m^2^)** | **EL(nm)** | **ƞ_c_(cd/A)** | **ƞ_p_(lm/W)** | **CIE(x,y)** | **ref** |
| --- | --- | --- | --- | --- | --- | --- | --- |
| **NDBNPIN:Ir(MDQ)_2_ (acac)** | **2.4** | **-** | **639** | **26.0** | **31.9** | **0.64,0.36** | **This work** |
| **DBTPIN:Ir(MDQ)_2_ (acac)** | **2.4** | **-** | **632** | **29.8** | **36.0** | **0.64,0.36** | **This work** |
| TPP | - | 42 | 655 |  | - | 0.70, 0.28 | 60 |
| TPC | - | 100 | 660 | - | 0.061 | 0.67, 0.29 | 61 |
| TPDPP | - | 150 | 635 | - | 0.035 | 0.69, 0.29 | 62 |
| ACY | - | 6400 | - | - | 1.3 | 0.68, 0.32 | 63 |
| CQY | - | 1000 | - | - | 0.28 | 0.70, 0.30 | 63 |
| NPAMLMe | - | 8000 | 650 | 1.5 | 0.9 | 0.66, 0.32 | 64 |
| BZTA2 |  | 9138 | 626 | 2.0 | 1.6 | 0.63, 0.35 | 65 |
| BZTA1 | - | 8087 | 640 | 0.91 | 0.58 | 0.63, 0.35 | 65 |
| INDMLMe | - | 1750 | 650 | - | - | 0.63, 0.36 | 66 |
| pAAA | - | - | 616 | 0.6 | - | 0.63, 0.36 | 67 |

**Supplementary Information Scheme, Figures and Table legends**

**SI-I: Scheme S1**. Synthetic route of NDBNPIN and DBTPIN.

**Figure S1.** (a) Lippert–Mataga plot; solvatochromic b) absorption and c) emission spectra of NDBNPIN and DBTPIN.

**Figure S2.** ^1^H NMR spectrum of 4-(2-(4-(4'-(diphenylamino)-[1,1'-biphenyl]-4-yl)naphthalen-1-yl)-1H-phenanthro [9,10-d]imidazol-1-yl)-1-naphthonitrile (NDBNPIN)

**Figure S3.** ^13^C NMR spectrum of 4-(2-(4-(4'-(diphenylamino)-[1,1'-biphenyl]-4-yl)naphthalen-1-yl)-1H-phenanthro [9,10-d]imidazol-1-yl)-1-naphthonitrile (NDBNPIN)

**Figure S4.** ^1^H NMR spectrum of 4-(2-(5-(4'-(diphenylamino)-[1,1'-biphenyl]-4-yl)thiophen-2-yl)-1H-phenanthro[9,10-d] imidazol-1-yl)-1-naphthonitrile (DBTPIN)

**Figure S5.** ^13^C NMR spectrum of 4-(2-(5-(4'-(diphenylamino)-[1,1'-biphenyl]-4-yl)thiophen-2-yl)-1H-phenanthro[9,10-d] imidazol-1-yl)-1-naphthonitrile (DBTPIN

**Figure S6.** MALDI-TOF mass spectra of (a) NDBNPIN and (b) DBTPIN

**Figure S7.** Natural transition orbital pairs with (HONTOs and LUNTOs) transition character analysis for singlet states (S_1_-S_5_) of NDBNPIN [*f*-oscillator strength and % weights of hole-particle].

**Figure S8.** Natural transition orbital pairs with (HONTOs and LUNTOs) transition character analysis for singlet states (S_1_-S_5_) of DBTPIN [*f*-oscillator strength and % weights of hole-particle].

**Figure S9.** Molecular structure, ground and excited state geometries with dihedral angles of NDBNPIN and DBTPIN.

**Figure S10.** Potential energy scan of (a) NDBNPIN and (b) DBTPIN; potential energy scan of excited state of NDBNPIN and DBTPIN (c&d) with increasing solvent polarity.

**Figure S11.** (a) Energy level diagram of singlet (S) and triplet (T) and (b) schematic diagram of hybridization processes of LE and CT states of NDBNPIN and DBTPIN.

**Figure S12.**(a) Transition density matrices (TDM) of NDBNPIN for [S_1_–S_3_ states] and (b) hole and particle distribution [S_1_–S_3_ states: green-increasing electron density and blue-decreasing electron density]

**Figure S13.** (a) Transition density matrices (TDM) of DBTPIN for [S_1_–S_3_ states] and (b) hole and particle distribution [S_1_–S_3_ states: green-increasing electron density and blue-decreasing electron density]

**Figure S14.** Computed difference in total density for ground and excited states [Δρ (r)=ρE_x_ (r)-ρG_s_ (r); isosurface for NDBNPIN, DBTPIN, (0.0000006 a.u) and graphical representation of DCT and centroid of charges [C+(r) / C-(r); isosurface for NDBNPIN (0. 29 a.u) and for DBTPIN (0.1 a.u)]

**Table S1**: Summary of device efficiencies with reported non-doped emitters.

**Table S2:** Summary of device efficiencies with reported green emitters.

**Table S3:** Summary of device efficiencies with reported red emitters.

**References**

1. Barford, W. Theory of singlet exciton yield in light-emitting polymers. *Phys. Rev. B.* **70**, 205204*,* DOI:https://doi.org/10.1103 (2004).

2. Jiang, W., Duan, L., Qiao, J., Dong, G., Wang, L., & Qiu, Y. Tuning of Charge Balance in Bipolar Host Materials for Highly Efficient Solution-Processed Phosphorescent Devices. *Org. Lett.* **13**, 3146 -3149, DOI: 10.1021/ol201039n (2011).

3. Tao, Y., Wang, Q., Yang, C., Zhong, C., Zhang, K., Qin, J., & Ma, D. Tuning the Optoelectronic Properties of Carbazole/Oxadiazole Hybrids through Linkage Modes: Hosts for Highly Efficient Green Electrophosphorescence. *Adv. Funct. Mater* **20***,* 304 -311, https://doi.org/10.1002/adfm.200901615 (2010).

4. Wang, B., Lv, X., Tan, J., Zhang, Q., Huang, Z., Yi, W., & Lei, W. Bipolar phenanthroimidazole–diazacarbazole hybrids with appropriate bandgaps for highly efficient and low roll-off red, green and blue electroluminescent devices. *J. Mater. Chem. C,* **4**, 8473-8482, DOI: 10.1039/C6TC02683D (2016).

5. Huang, T. H., Lin, J. T., Chen, L. Y., Lin, Y. T., & Wu, C. C. Dipolar Dibenzothiophene S,S-Dioxide Derivatives Containing Diarylamine: Materials for Single-Layer Organic Light-Emitting Devices. *Adv. Mater.* **18,** 602-606, https://doi.org/10.1002/adma.200502078 (2006).

6. Ma, Z., Wang, E., Jarvid, M. E., Henriksson, P., Inganas, O., Zhang, F., & Andersson, M. R. Synthesis and characterization of benzodithiophene–isoindigo polymers for solar cells. *J. Mater. Chem*. **22**, 2306 -2314, DOI: 10.1039/C1JM14940G (2012).

7. Bulovic, V., Shoustikov, A., Baldo, M. A., Bose, E., Kozlov, V. G., Thomoson, M. E., & Forrest, S. R. Bright, saturated, red-to-yellow organic light-emitting devices based on polarization-induced spectral shifts. *Chem. Phys. Lett*. **287**, 455-460, https://doi.org/10.1016/S0009-2614(98)00168-7 (1998).

8. Zhang, S., Dai, Y., Luo, S., Gao, Y., Gao, N., Wang, K., Zou, B., Yang, B., & Ma, Y. Rehybridization of Nitrogen Atom Induced Photoluminescence Enhancement under Pressure Stimulation. *Adv.Funct. Mater.* **27**, 1602276, https://doi.org/10.1002/adfm.201602276 (2017).

9. Kim, R., Lee, S., Kim, K. H., Lee, Y. J., Kwon, S. K., Kim, J. J., & Kim, Y. H. Extremely deep blue and highly efficient non-doped organic light emitting diodes using an asymmetric anthracene derivative with a xylene unit. *Chem. Commun.* **49**, 4664-4666, DOI: 10.1039/C3CC41441H (2013).

10. Fan, C., Chen, Y. H., Jiang, Z. Q., Yang, C. L., Zhong, C., Qin, J. G., & Ma, D. G. Diarylmethylene-bridged triphenylamine derivatives encapsulated with fluorene: very high Tg host materials for efficient blue and green phosphorescent OLEDs. *J. Mater. Chem*. **20**, 3232 -3237, DOI: 10.1039/B927576B (2010).

11. Chou, P. Y., Chou, H. H., & Chen, Y. H. Efficient delayed fluorescence via triplet–triplet annihilation for deep-blue electroluminescence. *Chem. Commun.* **50**, 6869-6871, DOI: 10.1039/C4CC01851F (2014).

12. Zhu, M. R., & Yang, C. L. Blue fluorescent emitters: design tactics and applications in organic light-emitting diodes. *Chem. Soc. Rev.* **42**, 4963-4976, DOI: 10.1039/C3CS35440G (2013).
